# Systematic discovery of motif-based interactions of the auxiliary domains of USP family deubiquitinases

**DOI:** 10.1038/s41467-026-73047-7

**Published:** 2026-05-18

**Authors:** Aimiliani Konstantinou, Alicia Córdova-Pérez, Julia K. Varga, Priyanka Madhu, Leandro Simonetti, Maximilian Vieler, Ryosuke Ishimura, Frederic Lamoliatte, Ora Schueler-Furman, Norman E. Davey, Yogesh Kulathu, Ylva Ivarsson

**Affiliations:** 1 Department of Chemistry for Life Sciences, Uppsala University, Box 576, Uppsala, Sweden; 2 MRC Protein Phosphorylation & Ubiquitylation Unit, Faculty of Life Sciences, University of Dundee, Dundee, UK; 3 Departments of Quantitative Molecular Medicine (QMM) & Department of Microbiology and Molecular Genetics, Institute for Biomedical Research IMRIC, Faculty of Medicine, Hebrew University of Jerusalem, Jerusalem, Israel; 4 Division of Cancer Biology, The Institute of Cancer Research (ICR), London, UK; 5 Department of Cell and Molecular Biology, St. Jude Children’s Research Hospital, Memphis, TN, US

**Keywords:** Biochemistry, Proteins

## Abstract

The ubiquitin-specific proteases (USPs) family is the largest family of human deubiquitinating enzymes (DUBs). While most USPs are agnostic to polyubiquitin linkage-type, their substrate specificity is thought to be mediated by the recognition of the ubiquitnated protein itself. In addition to their catalytic domain, USPs have one or more auxiliary domains (ADs) with key functions in regulating DUB activity and localization. We hypothesize that some ADs bind short linear motifs (SLiMs) typically found in intrinsically disordered regions of proteins to achieve targeting to substrates and multiprotein complexes. To test this, we systematically assess the potential of 29 USP-ADs and two full-length USPs for SLiM binding using a combination of proteomic-peptide phage display, peptide SPOT arrays and affinity measurements. We discover SLiM-based interactions for 14 ADs from 9 USP-DUBs, including CYLD, USP11, USP19, USP20, USP22 and USP33, and define the consensus motif and properties of the SLiM-AD binding. Interestingly, we establish that the zf-UBP and DUSP2 domains of USP20 and USP33 are SLiM binding ADs with similar binding profiles, explaining the functional redundancy between the two DUBs. Our work reveals unique motifs recognized by the auxiliary domains CAP-Gly, UBL, zf-UBP and DUSP, with potential functional implications for substrate recognition and complex assemblies.

## Abstract (web summary)

USP deubiquitinases rely on auxiliary domains, yet their motif-binding is poorly defined. Here, the authors screen 29 auxiliary domains, characterize binding motifs, and reveal shared binding by USP20/USP33, with potential implications for substrate recognition and complex assembly.

## Introduction

Approximately 100 human deubiquitinating enzymes (DUBs) contribute to controlling the ubiquitination status of the cell^1^. They are responsible for removing ubiquitin from a vast number of ubiquitination sites by cleaving either the covalent bond between ubiquitin and the substrate or within polyubiquitin chains. Substrate specificity of DUBs is often achieved by recognition of specific ubiquitin linkage types or ubiquitin chain length^2,3^. However, many ubiquitin-specific proteases (USPs) demonstrate promiscuity in recognizing linkage types, rather than demonstrating specificity for the substrate that is ubiquitnated.

With 58 members, the USP family represents the largest human family of DUBs. Most USPs possess a modular architecture featuring auxiliary domains (ADs) in addition to their catalytic core that enable substrate recognition independently of the linkage type. Some of the ADs have been shown to bind to short linear motifs (SLiMs), which are typically 3–10 amino acid stretches located within intrinsically disordered regions (IDRs)^4,5^. These interactions may contribute to substrate targeting or complex assembly. A well-known example is USP7, which has an N-terminal MATH (meprin and TRAF homology) domain and a UBL (ubiquitin-like) domain that bind to distinct SLiMs ([PAE]xxS and KxxxK, respectively)^6–8^. Another example is USP8, which features an N-terminal MIT (Microtubule-Interacting and Trafficking) domain that binds to an MIT-interacting motif 1-like SLiM ([DE][LIF]x{2,3}R[FYIL]xxL[LV]) found in ESCRT-III complex proteins and targets the protein to endosomes^9,10^. USP8 also has a catalytically inactive rhodanese domain recently found to engage in SLiM-based interactions with potential substrates^11^. Other USPs with reported SLiM-binding ADs include the cylindromatosis protein (CYLD), which has three cytoskeleton-associated protein-glycine-rich (CAP-Gly) domains of which one reportedly binds to a proline-rich peptide in NEMO (NF-kappa-B essential modulator; also called IKK-gamma)^12^ and USP11, which has a SLiM-binding tandem DUSP (domain present in USPs)-UBL domain^13^. Additionally, the zf-UBP (zinc finger-ubiquitin binding protein) domains of USP5 and USP16 bind to the C-terminal diglycine motif of ubiquitin^14,15^. Despite these emerging insights, the SLiM-binding potential of ADs of most human DUBs remains largely uncharacterized. Uncovering these interactions could provide a deeper understanding of how DUBs achieve substrate specificity, coordinate with cellular complexes, and regulate diverse signaling pathways through motif-guided recognition.

In this study, we systematically screen for SLiM-binding of 29 ADs from 19 USPs and two full-length USPs (Suppl. Data 1). Proteins are screened against a peptide-phage library that tiles the intrinsically disordered regions of the human proteome (called HD2)^16^. We identify potential peptide ligands for 15 of the protein baits. For 9 of the domains, we define consensus binding motifs through an integrated approach combining ProP-PD data, structural modeling, and peptide SPOT array alanine scanning. Most (7 out of 9) of these consensus motifs were not defined before this study. Notably, for USP20 and USP33, we discover that both their zf-UBP and DUSP2 domains act as peptide-binding modules, each capable of interacting with a wide range of peptides. Moreover, many of the interacting proteins and potential substrates contain multiple candidate SLiM-binding sites, suggesting a putative cooperative mode of interaction. Collectively, our findings uncover previously unrecognized peptide-binding properties across several USP ADs and point to broader functional and regulatory roles for SLiM-mediated interactions in the context of deubiquitination.

## Results

### Identification of peptide ligands of auxiliary domains of USPs

**Fig. 1:**
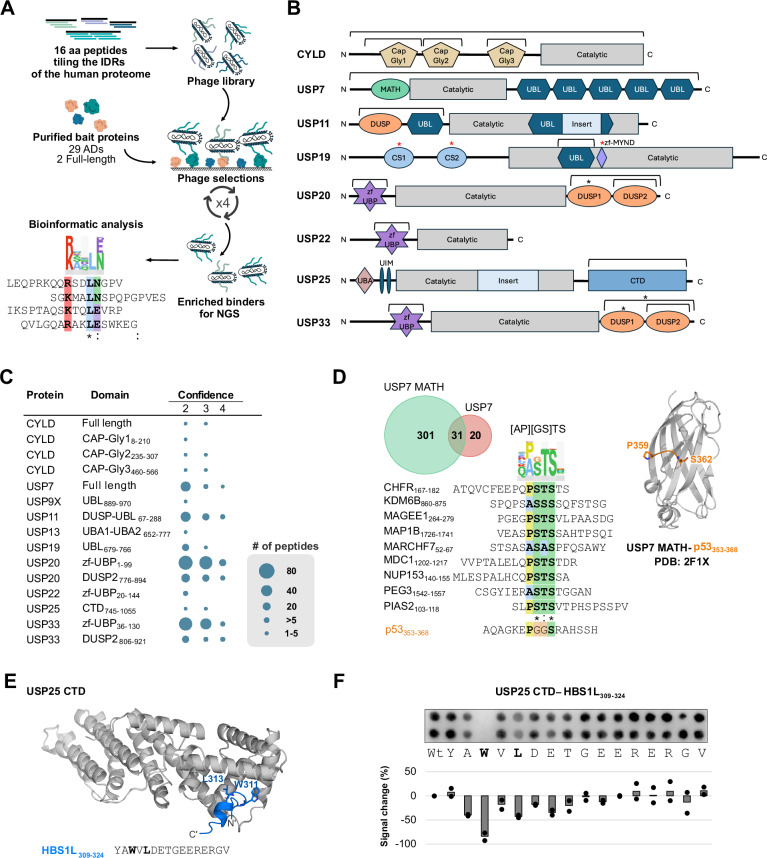
Overview of the ProP-PD selection results for auxiliary domains of DUBs and the full-length CYLD and USP7, including examples of DUBs binding to peptides and motifs. **A** Overview of the ProP-PD selections. **B** Schematic representation of the domain composition of the DUBs investigated. The protein or protein domains that were successfully produced and used as baits in phage selections are indicated by square brackets. Domains that were screened without enrichment of binding phages are marked with a red asterisk. Domains that could not be purified are marked with a black asterisk. **C** Overview of the ProP-PD selection results. The number of peptides enriched for each bait with given confidence (4 = highest quality data) is proportional to the area of the circle. **D** Peptide binding of USP7. Left: Overlap between previous ProP-PD data for the USP7 MATH domain^16^ and the results obtained for the full-length USP7 in the current study is depicted by a Venn diagram. Position-specific scoring matrix (PSSM) and alignment of the representative USP7 binding peptides found in this study are shown (the asterisk indicates full conservation and the semicolon semi-conserved residues). The p53 peptide is shown for comparison. Right: Structure of the USP7 MATH domain binding to the PxxS-containing p53 peptide (PDB: 2F1X^6^). **E** Peptide binding of the C-terminal domain of USP25. AF3^18^ model of USP25 CTD binding to a peptide from HSB1L_309-324_ suggests a WxL motif that docks into a pocket of the domain (ipTM: 0.6). **F** Validation of the USP25 binding WxL motif by alanine scanning peptide SPOT array of the HSB1L_309-324_ peptide. Residues involved in binding are shown in bold. Signal intensities were normalized to wild-type (Wt) (individual points) and presented as average percent signal change (bar). Source data for this and all subsequent peptide SPOT arrays are provided in the Source Data file.

We generated a collection of 29 USP ADs and two full-length USPs (CYLD and USP7). The collection was biased towards domains that are present in several USPs, such as zf-UBP domains, UBL domains and DUSP domains (Suppl. Data 1). We also included ADs only found in specific USPs, such as the three CAP-Gly domains of CYLD and the C-terminal domain of USP25. The proteins were used as baits in ProP-PD selections against the previously described HD2 library (Fig. 1A), which displays overlapping 16-amino-acid peptides tiling the intrinsically disordered regions of the human proteome flanked by glycine-serine linkers (SSSG- 16 aa peptide-GGGSGG)^16^. The peptide-coding regions of the phage pools were analyzed by next-generation sequencing (NGS), mapped to the corresponding proteins, and filtered for high/medium confidence ligands based on established criteria (with confidence level 4 being the highest). In total, we found 502 peptides binding to 14 bait protein domains and the full-length proteins CYLD and USP7 (Fig. 1B, C, Suppl. Data 2).

Analyzing the peptides using the SLiMFinder algorithm^17^ revealed consensus motifs for full-length USP7, USP11 DUSP-UBL, USP20 zf-UBP and DUSP2, and USP33 zf-UBP and DUSP2. Among them, full-length USP7 preferentially binds to a [AP][GS]TS motif (Fig. 1D). This motif matches the consensus USP7 MATH domain binding motif, found for example in the tumor suppressor p53, a bona fide substrate of USP7^6^ (Fig. 1D). We previously used the USP7 MATH domain as bait in ProP-PD selections and validated that it binds to a [AP][GS]xS motif^16^. Here, we found 51 ligands, of which more than 60 % overlap with those identified for the isolated USP7 MATH domain (Fig. 1D). The identified peptide ligands were consistently modeled to bind to the MATH domain binding pocket when using either the isolated MATH domain or the full-length USP7 for AlphaFold3 (AF3) modeling^18^ (Supplementary Fig. 1A, B). Our screen unraveled binding sites of the USP7 MATH domain in several previously reported USP7 interactors, such as the lysine-specific demethylase 6B (KDM6B), the mediator of DNA damage checkpoint protein 1 (MDC1), and the E3 ubiquitin-protein ligases CHFR and MARCHF7^19–22^ (Fig. 1D). CHFR and MDC1 are also previously validated USP7 substrates^22,23^. The USP7 results highlight the robustness of the ProP-PD approach in identifying relevant interactions.

For the eight ADs from CYLD, USP9X, USP13, USP19, USP22 and USP25, we identified limited sets of peptide ligands and no clear consensus motifs. Nevertheless, some of these peptides provided valuable starting points for motif discovery. For example, the C-terminal domain (CTD) of USP25 enriched a peptide region from the HBS1-like protein (HBS1L) spanning residues 305–324 (ASFAYAWVLDETGEERERGV, with the underlined stretch appearing in two overlapping peptides). An AF3^18^ model of the USP25 CTD–HBS1L interaction (ipTM: 0.6) suggests that the WxL motif docks into a pocket formed by the N-terminal helices of the domain (Fig. 1E, Supplementary Fig. 1C). This interaction was further validated using a peptide alanine-scanning peptide array, which confirmed the importance of the WxL motif for binding (Fig. 1F). These findings illustrate how even a limited number of peptide hits can be mined to reveal previously unrecognized, motif-driven interactions.

### SLiM-based interactions of the CYLD CAP-Gly domains

**Fig. 2:**
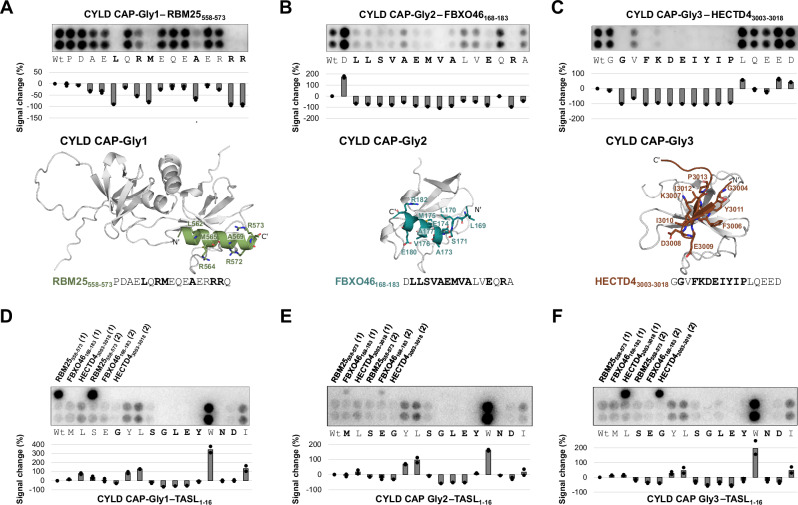
The three CAP-Gly domains of CYLD are peptide binding domains. **A** Peptide binding of CYLD CAP-Gly1. Alanine scanning peptide SPOT array (top) confirms an interaction between CYLD CAP-Gly1 domain and the RBM25_558-573_ peptide and identifies residues critical for binding. The AF3 model of the complex (bottom; ipTM: 0.93) suggests that the peptide forms an α-helix upon binding and supports that the identified residues are making contacts with the domain. **B** Peptide binding of the CAP-Gly2 domain of CYLD. Alanine scanning peptide SPOT array (top) confirms an interaction between CYLD CAP-Gly2 and the FBXO46_168-183_ peptide. The AF3 model of the complex (bottom; ipTM: 0.8) suggests that the peptide forms an α-helix upon binding. **C** Peptide binding of the CAP-Gly3 domain of CYLD. Alanine scanning peptide SPOT array (top) confirms an interaction between CYLD CAP-Gly3 and the HECTD4_3003-3018_ peptide, and that the interaction is sensitive to mutations of an extended stretch. The AF3 model of the complex (bottom; ipTM: 0.78) suggests that the peptide forms a β-hairpin structure upon binding. **D–F** Alanine scanning peptide SPOT array analysis of the TASL peptide binding to the CYLD CAP-Gly1 (**D**), CAP-Gly2 (**E**) and CAP-Gly3 (**F**) domain. Peptides from panel A-C used as positive and negative controls for each domain. **A–F** Signal intensities were normalized to wild type (Wt) (individual points) and presented as average percent signal change (bar). Source data are provided in the Source Data file.

CYLD is a tumor suppressor that regulates the stability of NF-κB and numerous other targets^24^. It has three CAP-Gly domains, a domain type which is known to interact with peptide ligands and typically recognize an acidic C-terminal motif defined as [ED]x{0,2}[EDQ]x{0,1} [YF]-COOH^25^ (Supplementary Fig. 2A). In CYLD, the CAP-Gly1 and CAP-Gly2 domains facilitate interactions with microtubules, while the CAP-Gly3 domain is known to bind a peptide from NEMO (also called IKKgamma)^12^. In addition, the CAP-Gly2 and CAP-Gly3 domains have been reported to bind to ubiquitin (Supplementary Fig. 2D)^26^. To identify peptide ligands binding to CYLD, we screened the individual CAP-Gly domains through ProP-PD and found one to four ligands per domain (Fig. 2A–C). We explored the interactions of the top hits of each domain using alanine scanning peptide arrays. The analysis validated the interaction of the CAP-Gly1 domain with a peptide from the RNA-binding protein 25 (RBM25), and showed it to bind via an atypical CAP-Gly binding motif: LxxMxxxAxxRR (Fig. 2A; called CG1IM). AF3 modeling (ipTM: 0.93) predicted that the peptide adopts an α-helical conformation upon binding the first CAP-Gly1 domain of CYLD (Fig. 2A). The CAP-Gly2 domain was confirmed to bind to a peptide from FBXO46 (F-box only protein 46; 168-DLLSVAEMVALVEQRA-183), and this interaction was found to be sensitive to mutations at most peptide positions (Fig. 2B). The interaction between CYLD CAP-Gly2 and the FBXO46 peptide was modeled with high confidence using AF3 (ipTM: 0.8) (Fig. 2B). Notably, similar to the CAP-Gly1 binding peptide, the CAP-Gly2 binding peptide was modeled in α-helical conformations, but oriented in the opposite direction (Fig. 2A, B). The CAP-Gly3 domain was confirmed to bind a peptide from the probable E3 ubiquitin-protein ligase HECTD4. Alanine scanning peptide array showed that a GxFKDEIYIP stretch was critical for binding (Fig. 2C). The HECTD4-derived peptide was confidently AF3 modeled binding to the CAP-Gly3 domain in a β-hairpin conformation (ipTM: 0.78; Fig. 2C). The binding site of the HECTD4 peptide on the domain partially overlaps with the peptide binding site suggested by NMR for the NEMO peptide^12^. The results suggest a conserved binding surface with potential adaptability depending on the peptide ligands.

To get additional information, we screened the full-length CYLD for peptide binding, which resulted in a set of 10 peptides. Of these, two highly enriched and overlapping peptides belong to TASL (TLR adapter interacting with SLC15A4 on the lysosome). Peptide array analysis revealed that all CAP-Gly domains bind to the TASL peptide (Fig. 2D–F, Supplementary Fig. 2E–G). Thus, the selection against the CYLD full-length protein enriched a peptide that can bind all the three CAP-Gly domains. For the CAP-Gly2 domain, the spot intensity was higher for the TASL peptide as compared to the FBXO46 peptide, potentially suggesting a higher affinity for the TASL peptide. In contrast, the RBM25_558-573_ and HECTD4_3003-3018_ peptides appeared to be better binders of the CAP-Gly 1 and 3 domains, respectively.

Alanine scanning revealed that the key residues for TASL peptide binding to the three domains converge on a GLEYxND stretch (Fig. 2D–F). Notably, a tryptophan to alanine (W13A) mutation of the wild-card position enhanced the binding to all three domains. Taken together, we find that all three CYLD CAP-Gly domains are peptide-binding ADs, being bound by distinct, as well as shared peptide ligands.

### SLiM-based interactions of the ubiquitin-like (UBL) domains of USP19 and USP11

**Fig. 3:**
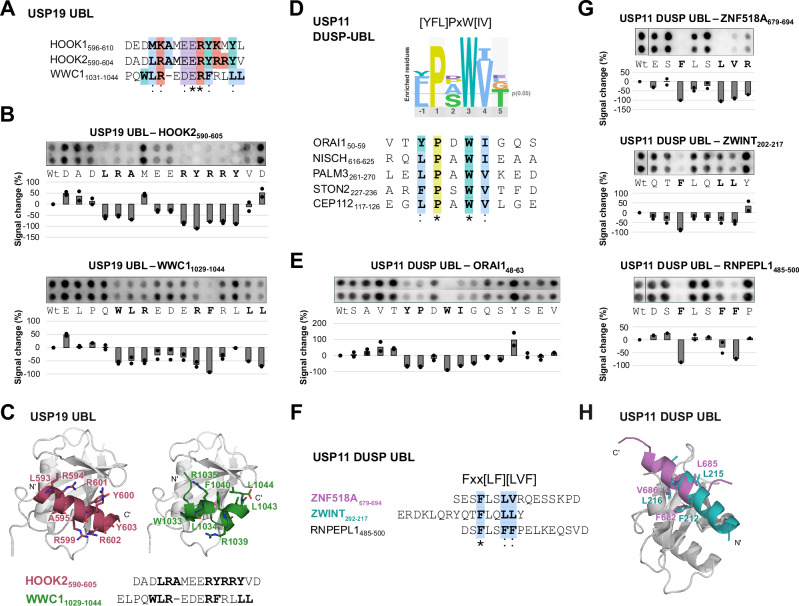
SLiM binding of the USP19 UBL and USP11 DUSP-UBL domains. **A** Alignment of the USP19 UBL binding peptides from HOOK2 and WWC1 identified through ProP-PD selections, together with the conserved region from the HOOK2 homologous protein HOOK1. The asterisk indicates full conservation and the semicolon semi-conserved residues. Additional USP19 UBL peptides are available in Suppl. Data 2. **B** Alanine scanning peptide SPOT array analysis of two USP19 UBL binding peptides from HOOK2_590-605_ and WWC1_1029-1044_. Signal intensities were normalized to wild type (Wt) (individual points) and presented as average percent signal change (bar). **C** AF3 models of the USP19 UBL domain with the HOOK2_590-605_ and WWC1_1029-1044_ peptides (ipTMs: 0.78 and 0.8, respectively). Key residues identified by alanine scanning are shown in stick representation. **D** Alignment of five USP11 DUSP-UBL domain binding peptides with an alternative [YFL]PxW[IV] motif, together with the generated PSSM. The asterisk indicates full conservation and the semicolon semi-conserved residues. **E** Alanine scanning peptide SPOT array of the interaction between USP11 DUSP-UBL and the ORAI1_48-63_ peptide. Signal intensities were normalized to wild type (Wt) (individual points) and presented as average percent signal change (bar). **F** Alignment of three USP11 UBL binding peptides from ZNF518A, ZWINT and RNPEPL1. Additional USP11 DUSP UBL binding peptides are available in Suppl. Data 2. The asterisk indicates full conservation and the semicolon semi-conserved residues. **G** Alanine scanning peptide SPOT array of the USP11 DUSP-UBL domain binding peptides ZNF518A_679-694_, ZWINT_202--217_ and RNPEL1_485-500_. Partial arrays are shown to highlight the identification of a common Fxx[LF][LVF] motif (see Supplementary Fig. 3C for the complete array results). Signal intensities were normalized to wild type (Wt) (individual points) and presented as average percent signal change (bar). **H** AF3 models of USP11 DUSP-UBL domain with the ZNF518A_679-694_ (ipTM: 0.7), ZWINT_202--217_ (ipTM: 0.71) peptides. The peptides are predicted to dock to the UBL domain. The motif key residues are shown in stick representation (see Supplementary Fig. 3E for a more detailed representation).

Ubiquitin-like (UBL) domains are homologous to ubiquitin and found in one third of the USP DUBs. Ubiquitin and some UBL domains are known to bind the ubiquitin interacting motif (UIM; [DE]x{2,4}[AV]xx[LMIV]xx) that forms an α-helix upon binding^27,28^ and variants thereof^29,30^. The USP11 tandem DUSP-UBL domain is also known to bind motifs using a distinct binding site^13^. To explore the potential SLiM mediated interactions of UBL domains of USP DUBs, we screened five UBL domains and identified binders for the UBL domain of USP19 and the tandem DUSP-UBL domain of USP11. For the USP19 UBL domain, the most enriched peptide was from the protein Hook homolog 2 (HOOK2) (Suppl. Data 2; Fig. 3A), a sequence that is conserved in the homologous protein, HOOK1. Peptide array alanine scanning analysis of a HOOK2 peptide (590-DADLRAMEERYRRYVD-605) and a peptide from the protein KIBRA (WWC1; 1029-ELPQWLREDERFRLLL-1044) identified partially shared binding determinants (LRx{3,4}R[FY]) (Fig. 3B) that are distinct from the typical UIM motif. AF3 modeling of the HOOK2 and WWC1 peptides bound to the USP19 UBL domain (ipTM 0.78 and 0.8, respectively) suggested that the peptides adopt α-helical conformations (Fig. 3C). Notably, the binding sites of the model peptides partially overlap with the UIM binding site of ubiquitin, although the sequence identities between the domains are low (Supplementary Fig. 3A) and the I44 in the central β-strand of the ubiquitin binding site, which is important for UIM binding^31^, is replaced by a valine in the USP19 UBL domain.

The USP11 DUSP-UBL tandem domain enriched for a diverse set of ligands, 17 of which contained a consensus [YFL]PxW[IV] motif (Fig. 3D). Alanine scanning analysis of a peptide from the calcium release-activated calcium channel protein 1 (ORAI1) (48-SAVTYPDWIGQSYSEV-63) confirmed the YPxWI motif (Fig. 3E). AF3 modeling of the DUSP-UBL ORAI1 complex resulted in low confidence models, likely reflecting a low affinity interaction, as determined by FP-based affinity measurements (K_D_: 200-300 μM; Supplementary Fig. 3B, Suppl. Data 3). Additionally, the selection against the USP11 DUSP-UBL tandem domain enriched another cohort of peptides with an apparent [LF]xx[LF][LVF] motif (Fig. 3F), which is similar to a previously characterized USP11 binding motif^13^. Key motif residues were confirmed in peptides from ZNF518A, ZWINT and RNPEPL1 by alanine scanning (Fig. 3G, Supplementary Fig. 3C). AF3 confidently modeled the ZNF518A and ZWINT peptides to a common site in the USP11 UBL domain (ipTM: 0.7 and 0.71, respectively,  Fig. 3H) which overlaps with a previously established USP11 UBL binding site for a AEGE**F**YK**L**KIRTPQ peptide (PDB: 5OK6; Supplementary Fig. 3D)^13^. The phenylalanine and the first leucine of the motif appear to be oriented towards the binding pocket (Supplementary Fig. 3E). Taken together, we found that the USP11 DUSP-UBL domain binds to two types of SLiMs, the previously described [LF]xx[LF][LVF] motif and the identified [YFL]PxW[IV] motif, representing an additional type of USP11 binding motif.

### The zf-UBP domains of USP20 and USP33 recognize a [KR]xxL[NED] motif

USP20 and USP33, also known as VHL-interacting DUBs hVDU2 and hVDU1, are ER-localized DUBs that regulate various cellular biological processes, such as cell cycle progression and proliferation^32–34^. USP20 and USP33 have a similar domain organization (Fig. 1B). They consist of four domains: an N-terminal zf-UBP domain, a catalytic domain, and two closely linked ubiquitin specific proteases 1 (DUSP1) and (DUSP2) domains (Fig. 1B). We screened the zf-UBP and DUSP2 domains of USP20 and USP33, as well as the DUSP1-DUSP2 tandem of USP20.

**Fig. 4:**
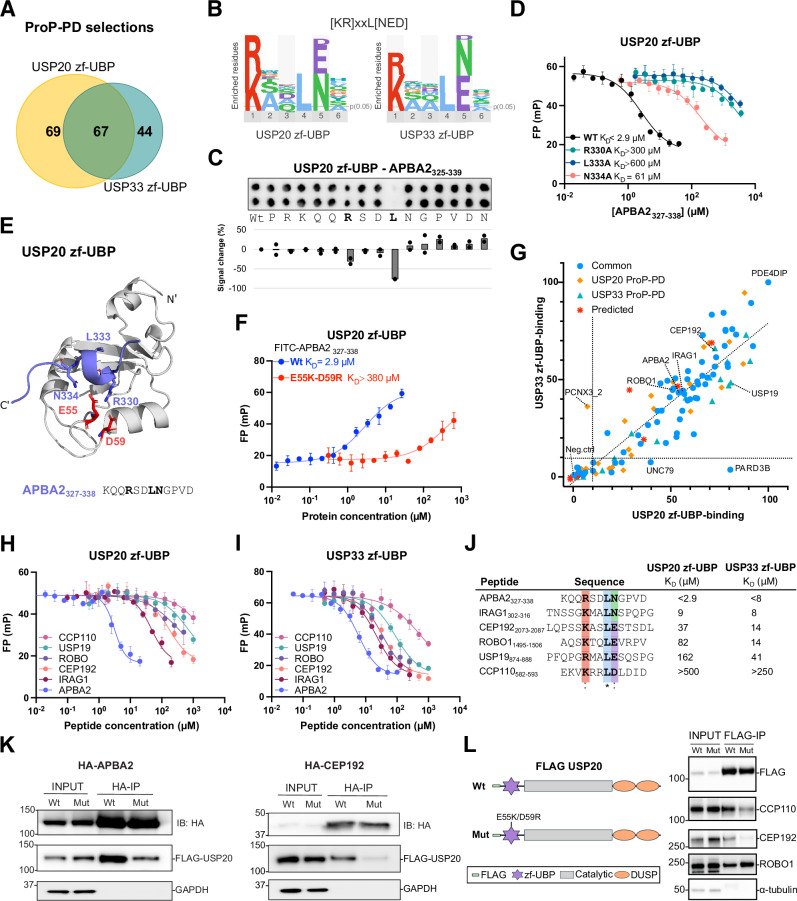
The zf-UBP domains of USP20 and USP33 bind to [KR]xxL[NED] motif containing peptides. **A** Venn diagram of the overlap of the peptide-binding regions identified as ligands for the zf-UBP domains of USP20 and USP33 through ProP-PD selections. **B** Consensus binding [KR]xxL[NED] motif for USP20 and USP33 zf-UBP domains. **C** Peptide SPOT array alanine scanning of the APBA2_325-339_ peptide binding to the USP20 zf-UBP domain. Signal intensities were normalized to wild type (Wt) (individual points) and presented as average percent signal change (bar). **D** Competitive FP-monitored affinity measurements of USP20 zf-UBP binding to wild-type and mutant APBA2_327-338_ peptides. Measurements were performed in technical triplicate, and the data are presented as means ± SD. Source data for these and all subsequent FP-monitored affinity measurements are provided in Source data files. **E** AF3 model of the interactions between the APBA2_327-338_ peptide and the zf-UBP domain of USP20 (ipTM: 0.87, pLDDT> 80 over the motif residues). **F** FP-monitored affinity measurements between wild-type and double mutant zf-UBP domains of USP20 and the APBA2_327-338_ peptide. Measurements were performed in technical triplicate, and the data are presented as means ± SD. The mutated domain residues are shown in (**E**). **G** Scatter plot of the normalized peptide SPOT intensities observed for 120 peptides against the zf-UBP domains of USP20 and USP33. The peptides used to design the array were found in phage selections against both USP20 and USP33 (“Common”), in either of the two USP20 and USP33 zf-UBP domain datasets, or were predicted ligands based on the consensus motif. The diagonal line represents the linear regression of the data (slope: 0.82). The dotted lines indicate a cut-off of 10% signal over background. Peptide sequences and peptide array analysis results are available in the Suppl. Data 4. **H–J**. Competitive FP-monitored affinity measurements of USP20 (**H**) and USP33 (**I**) zf-UBP domains binding to six distinct peptides, together with the estimated K_D_ values (**J**). Measurements were performed in technical triplicate, and the data are presented as means ± SD. Alignment of the binding peptides identified through ProP-PD selections. The asterisk indicates full conservation and the semicolon semi-conserved residues. **K** Co-immunoprecipitation experiments of FLAG-USP20 with wild-type HA-APBA2 and mutant (R330A/L333A) and CEP192 K1743-K2092 and mutant (K2078A/L2081A) in HEK293 cells (repeated in at least two independent experiments). Original blots for these and all subsequent Western blot experiments are provided in Source data files. **L** Immunoprecipitation of wild-type USP20 and predicted zf-UBP binding pocket mutants (repeated in at least two independent experiments). Left: Schematic representation of the USP20 zf-UBP predicted binding pocket mutants (E55K/D59R). Right: Immunoprecipitation experiments of the FLAG-USP20 with endogenous CCP110, CEP192 and ROBO1.

For the zf-UBP domains we identified 136 and 111 peptide-binding regions, respectively, with an overlap of 67 peptides binding both domains (Fig. 4A). Based on the identified peptides, consensus motifs were generated using the SLiMFinder algorithm^17^ which converged on a [KR]xxL[NED] motif, here called UBPIM (zf-UBP interacting motif; Fig. 4B). Interestingly, for many of the ligands, ubiquitination sites are present in and around the UBPIMs (Suppl. Data 2). To validate the importance of each residue in this motif to zf-UBP binding, we performed an alanine scanning analysis of a peptide (325-PRKQQ**R**SD**LN**GPVDN-339) from amyloid-beta A4 precursor protein-binding family A member 2 (APBA2; also known as MINT2), a previously reported interactor of USP20/USP33 identified in affinity-purification mass spectrometry (AP-MS) and yeast two-hybrid (Y2H) experiments^35–37^. The analysis confirmed binding and established a key requirement for leucine at the 4^th^ position of the UBPIM (Fig. 4C). Furthermore, an R to A mutation of the first position of the motif affected the interaction. To further assess the motif-determinants we conducted FP-monitored affinity measurements of wild-type and mutant APBA2_327-338_ peptides against the zf-UBP domains of USP20 and USP33 (Fig. 4D; Supplementary Fig. 4A). While the wild type APBA2 peptide bound with high affinity (USP20 K_D_ < 2.9 μM; USP33; K_D_ < 8 μM), mutation of the arginine at the first position of the motif (R330A; USP20: K_D_ > 300 μM, USP33: K_D_ > 500 μM) and the leucine at position 4 (L333A; USP20: K_D_ > 600 μM, USP33: K_D_ = ND) significantly weakened binding (Fig. 4D, Supplementary Fig. 4A, Suppl. Data 3). Additionally, the asparagine at position 5 contributes to binding, as revealed by a N334A mutant conferring reduced affinity for USP20 and USP33, albeit to less extent (USP20 K_D_ = 61 μM; USP33 K_D_ = 112 μM) (Fig. 4D; Supplementary Fig. 4A). Taken together, the USP20/33 zf-UBP interaction is centered on the [KR]xxL[NED] motif.

AF3 docking of the APBA2_327-338_ peptide with USP20/USP33 zf-UBP domains resulted in high-confidence models (Supplementary Fig. 4B; USP20 ipTM: 0.87, USP33 ipTM: 0.83, pLDDT> 80 over the motif residues). The UBPIM binding pocket of these domains overlaps with the binding site for C-terminal ubiquitin present in other zf-UBP domains, such as USP5 and USP16^15,38^ (Supplementary Fig. 4C). In contrast to most other zf-UBP domains, the USP20 and USP33 zf-UBP domains do not bind the ubiquitin C-terminus with measurable affinity (Supplementary Fig. 4D). Nevertheless, the interactions between the APBA2_327-338_ peptide and the USP20/USP33 zf-UBP domain (Fig. 4E, Supplementary Fig. 4E), have some resemblance to how the USP5 zf-UBP binds to the ubiquitin C-terminus (PDB: 2G45)^15^. Residue E55 in USP20 zf-UBP (E86 of USP33) interacts with the arginine at the first position of the [KR]xxL[NED motif and forms an additional salt bridge with USP20 zf-UBP D59 (D90 in USP33) (Fig. 4E, Supplementary Fig.4E). To validate the peptide binding site, we created double mutants of the USP20 and USP33 zf-UBP domains (E55K/D59R and E86K/D90R, respectively), and affinity measurements confirmed that the mutations significantly impair peptide binding (Fig. 4F, Supplementary Fig. 4E).

To assess the binding of a larger set of USP20/USP33 UBPIM ligands, we designed an array that included peptides that bind to both USP20 and USP33 zf-UBP domains based on the phage selection results (Fig. 4G, Suppl. Data 4). In addition, the array design included “unique” peptides only found as ligands for either of the zf-UBP domains. An additional set of nine predicted ligands (based on PSSMSearch^39^) were included. More than 80% of the peptides were found to bind to USP20, while over 70% of the peptides bind to the USP33 zf-UBP, with a signal intensity exceeding 10% relative to the background (Fig. 4G; Supplementary Fig. 4G). Applying a more stringent cut-off (20% signal over background), 79 ligands were confirmed to bind to one or both domains. Four of the nine predicted ligands were confirmed to bind the zf-UBP domains, suggesting that USP20/33 zf-UBP binding peptides can be predicted using the motif determinants with some accuracy. Importantly, most of the peptides bound to both domains, with a peptide from the partitioning defective 3 homolog B protein (PARD3B) (171-TQNLEDREVLNGVQTE-186) being an exception, as it only bound to USP20.

Amongst the validated ligands, we note a peptide from Roundabout homolog 1 (ROBO1_1495-1506_; see Supplementary Fig. 4F for AF3 model). ROBO1 is known to be deubiquitinated by USP33 in the Slit-ROBO signaling pathway and regulates cell migration^40,41^. The region of interaction between ROBO1 and USP33 has previously been mapped to the C-terminal stretch of ROBO1, which overlaps with the peptide discovered here. We found that the ROBO1_1495-1506_ peptide binds with almost six-fold higher affinity to USP33 zf-UBP over the USP20 zf-UBP domain, with K_D_ values of 14 μM and 82 μM, respectively (Fig. 4H–J). We further determined the affinities for a set of ProP-PD-derived ligands and one additional predicted binding peptide from the centriolar coiled-coil protein of 110 kDa (CCP110) (Fig. 4H–J). CCP110 is involved in centrosome duplication and is deubiquitinated and stabilized by USP33 during the S and G2/M phases of the cell cycle^42^. The affinities were in the range of low- to high-micromolar, with the affinity ranking of the peptides generally being the same for both domains. While the APBA2 peptide binds with the highest affinity (USP20: K_D_ < 2.9 μM, USP33: K_D_ < 8 μM) to both USP20 and USP33 zf-UBP domains, the predicted CCP110_582-593_ peptide was found to bind with low affinity.

To assess the binding of the UBPIM motif in the context of full-length proteins, we co-expressed FLAG-tagged USP20 with either wild-type or motif-mutant HA-tagged APBA2 and CEP192 (the centrosomal protein of 192 kD, amino acid region 1743-2092) and conducted co-IP experiments, which validated interactions with USP20 (Fig. 4K). The interactions were significantly reduced when the [KR]xxL[NE] motif was mutated, as seen for the R330A/L333A mutant HA-APBA2 and the K2078A/L2081A mutant HA-CEP192_1743-2092_ (Fig. 4K). We further generated binding pocket mutants of the zf-UBP in the context of full-length USP20 and assessed the effects on binding of FLAG-USP20 to endogenous CCP110, CEP192, and ROBO1 through immunoprecipitation experiments (Fig. 4L). As expected, mutations in the zf-UBP domain of USP20 reduced its interaction with CCP110 and CEP192. However, the mutations did not affect its interaction with ROBO1, which may suggest that the interaction involves additional binding determinants.

Taken together, we established that the zf-UBP domains of USP20 and USP33 are peptide-binding modules with largely overlapping specificities for the UBPIM-containing ligands, and that the motif is of importance for the interactions between the full-length proteins.

### The DUSP2 domains of USP20 and USP33 bind a [FW]x[IL] consensus motif

**Fig. 5:**
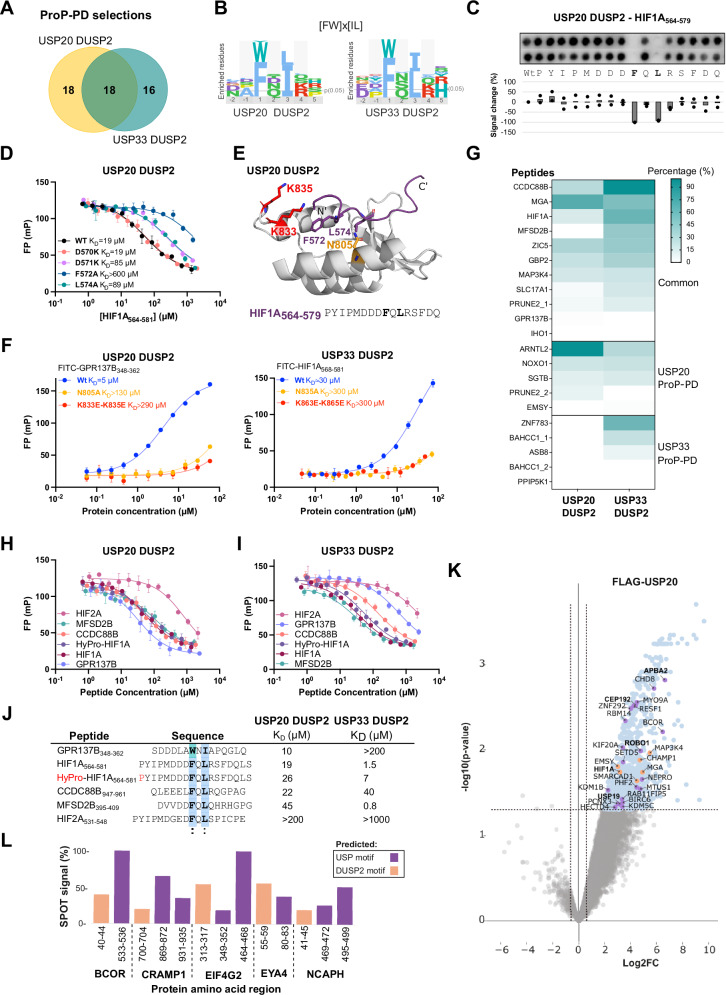
The DUSP2 domains of USP20 and USP33 bind [FW]x[IL] motif containing peptides. **A** Venn diagram of the overlap of USP20 and USP33 DUSP2 binding peptide-binding regions identified through ProP-PD selections. **B** Consensus binding motifs for USP20 and USP33 DUSP2 domains generated based on the peptide ligands identified through ProP-PD selections. **C** Peptide SPOT array alanine scanning of the HIF1A_564-579_ peptide binding to the USP20 DUSP2 domain. Signal intensities were normalized to wild type (Wt) (individual points) and presented as average percent signal change (bar). The experiment was performed in technical duplicate. **D** Competitive FP-monitored affinity measurements of USP20 DUSP2 binding to wild-type and mutant HIF1A_564-581_ peptides. Measurements were performed in technical triplicate, and the data are presented as means ± SD. **E** AF3 model of USP20 domain binding to HIF1A_564-579_ (ipTM: 0.7). The mutated domain residues are shown in (**E**). **F** Competitive FP-monitored affinity measurements of wild-type, single, and double mutant USP20 and USP33 DUSP2 domains binding to FITC-labeled [FW]x[IL] containing HIF1Aprobe peptides. Measurements were performed in technical triplicate, and the data are presented as means ± SD. **G** Heatmap representation of peptide SPOT array analysis of 21 peptides binding to USP20 and USP33 DUSP2 domains. Peptides were found in phage selections against both USP20 and USP33 (“Common”), or in either of the two USP20 and USP33 DUSP2 domain datasets. Peptide sequences and peptide array results are available in the Suppl. Data 5. **H–J** FP-monitored competitive affinity measurements of USP20 (**H**) and USP33 (I) DUSP2 domains binding to six peptide ligands, and the estimated K_D_ values (**J**). Measurements were performed in technical triplicate, and the data are presented as means ± SD. Alignment of the binding peptides identified through ProP-PD selections. The semicolon indicates semi-conserved residues. **K** FLAG-USP20 IP-MS analysis compared to the empty pCMV vector (Suppl. Data 6). The data were analyzed using the LIMMA framework (empirical Bayes moderated t-statistics; R package limma v4.4.3), and a two-sided statistical test. Resulting P-values were adjusted for multiple comparisons using the Benjamini–Hochberg false discovery rate correction. Overlap with ProP-PD-derived ligands is indicated in purple (zf-UBP) and orange (DUSP2). Interactors validated through FP-monitored affinity measurements are indicated in bold. MS source data are accessible in the PRIDE portal (Project accession: PXD068553). **L** Summary of peptide SPOT array validation of predicted dual UBPIM (purple) and DUSPIM motifs (orange) found in IP-MS-derived interactors. Peptide sequences and additional information for the predicted peptides are available in Suppl. Data 7. The peptide array membranes were incubated with the respective domains of USP20.

The two consecutive C-terminal DUSP domains (DUSP1 and DUSP2) of USP20 and USP33 have been suggested to play a role in substrate recognition^43^. ProP-PD screens of the DUSP2 domain and the the DUSP1-DUSP2 domains of USP20, identified 36 and 38 peptide binders respectively, with 21 common ligands (Supplementary Fig. 5A). For the USP33 DUSP2 domain, we identified 34 ligands, of which 18 also bind to the USP20 DUSP2 domain (Fig. 5A). The DUSP2 binding peptides share a [FW]x[IL] motif, here called DUSPIM (DUSP2 interacting motif; Fig. 5B). To validate the consensus motif, we focused on a peptide from the hypoxia-inducible factor 1-alpha (HIF1A), which is a previously reported USP20 substrate^44^. Peptide array alanine scanning confirmed the requirement for a F572 and a L574 in the DUSPIM (Fig. 5C). The importance of the motif residues was further assessed by FP-based affinity measurements, which showed that the HIF1A peptide binds to the DUSP2 domains of USP20 (K_D_ = 19 μM) and USP33 (K_D_ = 1.5 μM) (Fig. 5D; Supplementary Fig. 5B, Suppl. Data 3) and that mutation of motif residues significantly impacted DUSP2 binding (F572A: USP20 K_D_ > 600 μM; USP33 K_D_ > 900 μM; L574A: USP20 K_D_ = 89 μM; USP33 K_D_ > 200 μM). We noticed that the HIF1A peptide contains an acidic stretch of residues immediately upstream of the [FW]x[IL] motif, from D571 (p-1 position relative to motif) to D569 (p-3). A charge-reverting point mutation (D571K) reduced the affinity for the DUSP2 domains, albeit not to the same extent as the core hydrophobic residues (D571K: USP20 K_D_ = 85 μM; USP33 K_D_ = 145 μM) (Fig. 4D). In contrast, a D570K had relatively minor effects on binding. We further noticed that a proline (P564), located just upstream of the DUSP2 binding region in HIF1A is hydroxylated under normoxia. HIF1A proline hydroxylation results in an interaction with the von Hippel-Lindau disease tumor suppressor (pVHL), which subsequently triggers the ubiquitination and degradation of HIF1A^45^. As USP20 counteracts the ubiquitination of HIF1A and stabilizes the protein^44^ we evaluated the effect of hydroxylation on DUSP2 binding but found it to have minor effects on affinities (USP20 K_D_ = 26 μM; USP33 K_D_ = 7 μM; Fig. 5H–J).

AF3 modeling of the HIF1A_564-579_ peptide binding to the DUSP2 domains of USP20 and USP33 suggested the [FW]x[IL] motif (USP20 ipTM:0.7, USP33 ipTM:0.7, pLDDT>90 values over the residues; Supplementary Fig. 5C) to bind in hydrophobic pockets created by F801, I868, F825, E829, W828 in USP20 and F831, I897, F855, E859, W858 in USP33 (Fig. 5E; Supplementary Fig. 5D). Additional electrostatic interactions are predicted to be mediated between the acidic residues in the HIF1A peptide and K806 and K833 of USP20 (R836 and K863 of USP33). Furthermore, the backbone of the HIF1A peptide is kept in place with H-bonds, partially by complementing a short β-strand of the DUSP2 domain. Similar interactions were predicted for a peptide from the integral membrane protein GPR137B (Supplementary Fig. 5E). The DUSP2 peptide binding site was confirmed using single and double mutations of USP20 (N805A and K833E/K835E) and USP33 (N835A, K863E/K865E) (Fig. 5F). We next confirmed the interaction between full-length USP20 with HIF1A (either in normoxia or hypoxia-mimicking conditions) and assessed the effects of mutating the motif (Supplementary Fig. 5F-H). However, the motif mutant (HA-HIF1A F572A-L574A) did not affect binding (Supplementary Fig. 5F). Similarly, mutation of the DUSP2 domain (N804A, K832E, K834E) did not abrogate binding, implying a more stable association (Supplementary Fig. 5G, H). Deletion of either the zf-UBP domain or the DUSP2 domain disrupted recognition of a modified, presumably ubiquitnated, form of HIF1A (Supplementary Fig. 5G, H), suggesting a multivalent mode of interaction.

We designed a peptide array containing 21 peptides to evaluate the binding of a larger set of DUSPIM-containing peptides to the DUSP2 domains. Eleven of the peptides had been identified as ligands for both domains in the phage selection, and five peptides were identified as unique ligands to each domain (Fig. 5G; Suppl. Data 5). Binding was confirmed for 16 out of 21 tested peptides to either domain, with some noticeable specificity differences. In particular, none of the USP33-specific peptides from ProP-PD bind to USP20 DUSP2. In line with this, affinity measurements using an additional set of peptides from the phage selections (GPR137B_348-362_, coiled-coil domain-containing protein 88B (CCDC88B_947-961_), sphingosine-1-phosphate transporter MFSD2B_395-409_) and a predicted DUSP2 binding peptide from endothelial PAS domain-containing protein 1, also known as HIF2A (531-PYIPMDGEDFQLSPICPE-548) (Fig. 5H-J) supported specificity differences between the domains (Fig. 5J). While the UPS20 DUSP2 domain bound most ligands tested with similar affinities, the USP33 DUSP2 domain displayed higher specificity. Also, both domains bound the predicted HIF2A ligand with low affinity.

To substantiate the results, we conducted IP-MS experiments using FLAG-tagged wild-type USP20 and USP20 variants with either the zf-UBP or the DUSP2 domains deleted, and similar experiments for USP33 (Suppl. Data 6). USP20 (and USP33) pulled down a large number of proteins compared to the negative control (empty vector), including many interactors identified in ProP-PD, such as APBA2, CEP192, ROBO1, USP19, and HIF1A (Fig. 5K, Supplementary Fig. 5I). Comparison of the results obtained for the full-length and zf-UBP domain mutant variants revealed that the interaction with APBA2 is dependent on the zf-UBP binding pocket (Supplementary Fig. 5J), consistent with the FLAG-IP results of other interactors (Fig. 4L). The deletion of either the zf-UBP or the DUSP2 domains resulted otherwise in surprisingly few changes of the overall interactome profile of USP20 (Suppl. Data 6), suggesting that the interactions of the full-length proteins may involve multiple components (e.g. motif binding of both the zf-UBP domain and the DUSP2 domain, and/or the catalytic domain). We therefore scanned the IP-MS identified USP20 interactors for putative UBPIMs and DUSPIMs using the SLiMSearch algorithm and found that 44% of the proteins contain putative binding motifs for both domains in their intrinsically disordered regions (Suppl. Data 7). In contrast, only 27% of the proteins contained neither of the motifs in their IDRs. We tested the binding of 30 and 16 of the predicted UBPIMs and DUSPIMs, respectively, using a peptide array analysis, which confirmed 50% of the predicted interactions and the presence of both UBPIMs and DUSPIMs in several target proteins (Fig. 5L). For example, we validated predicted UBPIMs and DUSPIMs in the BCL-6 corepressor (BCOR), in the protein phosphatase EYA4, and the eukaryotic translation initiation factor 4 gamma 2 (EIF4G2).

Taken together, we uncover that both the zf-UBP and DUSP2 domains of USP20 and USP33 are SLiM-binding domains, which may contribute to complex assembly and substrate recognition. The results suggest that multiple components may act together to recruit USP20 and USP33 to their substrates. Further investigations are needed to explore the possibility of a cooperative substrate recognition mechanism.

### The zf-UBP domain of USP22 engages in SLiM-based interaction for complex assembly

**Fig. 6:**
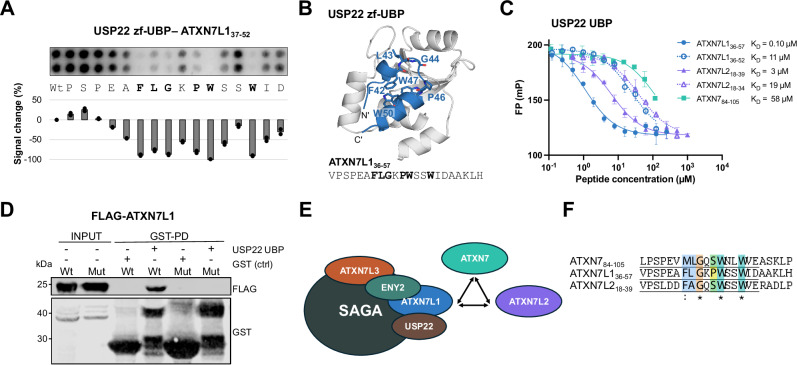
SLiM-based interactions of the USP22 zf-UBP domain. **A** Peptide SPOT array alanine scanning of the USP22 zf-UBP binding peptide from ATXN7L1_37-52_. Signal intensities were normalized to wild type (Wt) (individual points) and presented as average percent signal change (bar). The experiment was performed in a technical duplicate. **B** AF3 model of the complex between the USP22 zf-UBP domain and the ATXN7L1 peptide (ipTM: 0.83). The peptide residues are shown in stick representation. **C** FP-monitored affinity measurements between the USP22 zf-UBP domain and peptides from ATXN7_84-105_, ATXN7L2_18-34_, ATXN7L2_18-39_, ATXN7L1_36-52_, and ATXN7L1_36-57_. Measurements were performed in technical triplicate, and the data are presented as means ± SD. **D** GST-pulldown validation of the interaction between the GST-tagged USP22 zf-UBP domain and FLAG-tagged ATXN7L1 wild-type or motif mutant (W47A/S48G/S49G/W50A) from HEK293T cell lysate (repeated in three independent experiments). **E** Schematic representation of the SAGA complex, in which the ATXN7/7L1/L2 proteins are interchangeable. Schematic inspired by Felicio et al. 2023^56^. **F** Sequence alignment of the USP22 zf-UBP binding ATXN7/7L1/L2 peptides (asterisk indicates full conservation and the semicolon semi-conserved residues).

To conclude our survey, we screened the remaining 10 zf-UBP domains found in USPs for peptide binding. Most of these did not enrich any binding peptides. However, we found a small but interesting set of peptide ligands for the zf-UBP domain of USP22 (Fig. 6). USP22 is an oncogene promoting cancer progression^46,47^ and acts on histone substrates as part of the multiprotein transcriptional co-activator SAGA (Spt-Ada-Gcn5 acetyltransferase) complex, as well as on non-histone substrates^48–50^. Five potential peptide ligands were found for the USP22 zf-UBP domain, of which a peptide from the Ataxin-7-like protein 1 (ATXN7L1; 36-VPSPEAFLGKPWSSWI-51) (Fig. 6A) appeared particularly relevant, as it is part of the deubiquitination module of the SAGA complex, and the USP22 zf-UBP domain is known to be crucial for interactions within the SAGA complex^50–52^. Alanine-scanning peptide array analysis of the ATXN7L1 peptide confirmed a key role of the tryptophans and revealed the contribution of upstream and downstream residues (FLGxPWxxW) (Fig. 6A). The USP22 zf-UBP-binding ATXN7L1_36-51_ complex was modeled by AF3 with high confidence (ipTM: 0.73). An extended version of the ATXN7L1 peptide (36-VPSPEAFLGKPWSSWIDAAKLH-57) was modeled with higher confidence (pLDDT> 90; ipTM: 0.83) (Fig. 6B). The model suggests that the two tryptophans (W47, W50) dock into a hydrophobic cavity located on the opposite side of the typical zf-UBP binding site for the di-Gly motif of ubiquitin (Fig. 6B). The affinity of the USP22 zf-UBP-ATXN7L1_36-52_ interaction was found to be 11 μM, as determined by FP-monitored affinity measurements (Fig. 6C, Suppl. Data 3). Using the longer ATXN7L1_36-57_ peptide, the affinity increased to 0.10 μM, demonstrating a significant contribution of the region C-terminal of the ProP-PD derived ATXN7L1_36-52_ peptide to binding. The interaction between GST-USP22 zf-UBP and FLAG-ATXN7L1 was confirmed through GST-pulldown assays, and the interaction was abolished by mutations of the binding motif (W47A/S48G/S49G/W50A) (Fig. 6D).

The paralogous proteins ATXN7 and ATXN7L2, which are also part of the deubiquitination module of the SAGA complex and are thought to be interchangeable, contain peptide regions with similar conserved GxxWxxW sequences (Fig. 6E, F). Additionally, the N-terminal region of ATXN7 has been shown to be necessary for incorporation into the DUB module of the SAGA complex^53^. Thus, we determined the affinities of the ATXN7_84-105_ and ATXN7L2_18-39_ peptides for the USP22 zf-UBP domain, and found them to bind USP22 zf-UBP with slightly lower affinities than the ATXN7L1_36-52_ peptide (ATXN7_84-105_ K_D_ = 58 μM, ATXN7L2_18-34_ K_D_ = 19 μM; Fig. 6C, Suppl. Data 3). Based on the alignment of the three ATXN7/L1/L2 peptides and the alanine scanning results, we define the USP22 zf-UBP binding motif (USP22 UBPIM) as [FM][AL]Gx[PS]WxxW. Using a longer ATXN7L2_18-39_ peptide, we further confirmed a contribution of the C-terminal extension to binding, as it increased the affinity of the USP22 zf-UBP-ATXN7L2 interaction more than six times (K_D_ = 3 μM) (Fig. 6F). Notably, the USP22 UBPIM is similar to the interaction between the zf-UBP domain of the USP22 yeast orthologue Ubp8, and the N-terminal region (residues 1-40) of the yeast orthologue of ATXN7/L1/L2 (SAGA associated Factor, 73 kDa (Sgf73)^54^. The interaction involves a 30-DSWKSLM-36 stretch, which corresponds to 45-KPWSSWI-51 in human ATXN7L1^55^.

In the context of its regulatory function of the SAGA complex, USP22 forms part of a heterotetrameric complex with ATNX7/7L1/L2, ENY2 and ATXN7L3^56^, and forms contacts with all three proteins (Fig. 6E). To explore if the found SLiM-domain interaction between USP22 zf-UBP-ATXN7L1 was feasible in the heterotetrameric complex, we modeled it using AF3. Reassuringly, the found SLiM-domain interaction was predicted to bind virtually identically in this complex as in the model of the isolated USP22 zf-UBP domain and ATXN7L1 peptide complex (Supplementary Fig. 6).

Thus, we conclude that the zf-UBP domain uses distinct peptide binding sites for substrate recognition and complex assembly.

## Discussion

In this study, we systematically investigated SLiM-based interactions involving two full-length USPs and 29 ADs from the USP family of DUBs. We uncovered a diverse set of binding motifs and provide insights into the peptide-binding capabilities of the ADs. Our results suggest that SLiM-binding is a relatively common function among these domains. These interactions may contribute to complex and potentially substrate recognition analogous to the recognition of degrons by E3 ligases.

Almost all of the found motifs were undefined before the current study (Table 1), and not previously reported in, for example, the ELM database^5^. The study thus opens doors for follow-up studies on the interactions identified here, as well as the use of the defined motifs for scanning for binding sites within the intrinsically disordered regions of interaction partners and putative substrates using, for example, SLiMSearch^39^. Given the high false positive rates of such motif-based predictions, the analysis should be paired with experimental validations (for example, of a computational and experimental pipeline see ref. ^57^). Here, we used such an approach for uncovering additional binding sites for the zf-UBP and DUSP2 domains of USP20 and USP33 (Figs. 4G and 5L).

**Table 1:** Overview of SLiMs (re)defined in the current study

Gene	Domain	SLiM	Motif name	Previously described
CYLD	CAP-Gly_1	LxRMxxxAxxRR	CYLD CG1IM	
USP7	MATH	[AP][GS]TS	Variant of DOC_USP7_MATH_1	^5,16^,
USP11	UBL	Fxx[LF][LVF]	USP11 UIM_1	^13^
USP11	UBL	[LFY]PxW[IW]	USP11 UIM_2	
USP19	UBL	LRx{3,4}R[FY]	USP19 UIM	
USP20/USP33	zf-UBP	**[**KR]xxL[NED]	USP20/33 UBPIM	
USP20/USP33	DUSP2	[FW]x[IL]	USP20/33 DUSPIM	
USP22	UBP	[FM][AL]Gx[PS]WxxWx	USP22 UBPIM	
USP25	CTD	WxL	USP25 CTDIM	

We explored the identified interactions using AF3 and validated the models by mutagenic analysis. Modeling domains and peptide fragments rather than full-length proteins have been shown to improve prediction accuracy in AlphaFold-based approaches^58,59^. Several of the domain–peptide interactions found here were modeled with high confidence using AF3, based on ipTM scores. The confidence of the models was even higher when using the actifpTM score ^60^, which focuses scoring on the structured interface while discounting disordered flanking regions (Supp. Table 8). However, the precise structural details of these interactions within the context of the full-length protein complexes remain to be established experimentally. It remains possible that additional contacts contribute to complex formation in the full-length proteins, or that the structural context of the identified motifs within the intact proteins may impede binding.

For certain ADs, such as the zf-UBP domain of USP22, we identified only a few ligands, yet these ligands appear biologically relevant in the context of the assembly of the SAGA complex^53^. It is striking that the USP22 zf-UBP binding ATXN7L peptide was picked up, as the interaction between the full-length proteins involves additional contacts. In contrast, other domains, including the zf-UBP domains of USP20 and USP33, interact with a large number of binding motifs across the proteome. This observation is consistent with their roles as broad-specificity DUBs^32–34^. Furthermore, we show that both CYLD and USP20/33 harbor more than one SLiM-binding domain. This suggests that efficient complex assembly and substrate targeting may rely on a multicomponent recognition strategy, involving SLiM-based interactions mediated by ADs in addition to polyubiquitin chain recognition by the catalytic domain. We propose that coincidence detection, i.e., the binding of the SLiM motif by the AD plus ubiquitin on the motif-containing protein, enables the specific recognition of ubiquitinated substrates. Hence, the DUB-SLiM interaction identified in this study can guide experiments to define the cellular pathways regulated by these DUBs.

Another intriguing finding is the overlap between several identified USP20/USP33 zf-UBP binding sites and known sites of ubiquitination (Supp. Table 2). This suggests multiple possibilities for the functional interplay between ubiquitin “writers” (E3 ligases) and “erasers” (DUBs). One plausible scenario is that motif binding to zf-UBP domains shields specific lysine residues from ubiquitination, thereby modulating substrate availability. Alternatively, these interactions may reflect a mechanism of processive deubiquitination. While these scenarios remain hypothetical, they point to sophisticated regulatory strategies that warrant further experimental investigation. Interestingly, Marcello Clerici and colleagues^61^ demonstrated that the DUSP domain of USP4 facilitates ubiquitin release following hydrolysis, enhancing catalytic efficiency. Furthermore, the USP4 DUSP domain works synergistically with the UBL domain to retain the ubiquinated substrate in the active site, preventing competition from other substrates. By analogy, we hypothesize that the DUSP2 domain of USP20, may similarly collaborate with the zf-UBP domain to confer substrate specificity towards HIF1A, which is a previously described interactor of USP20. Other modes of regulation are of course possible, including phosphorylation sites overlapping with the binding motifs or located in the flanking regions, which may indicate further regulatory control.

Finally, several SLiM-binding DUBs are associated with human disease, reflecting their central roles in regulating protein stability via deubiquitination across multiple signaling pathways. For example, mutations in CYLD and USP7 are linked to familial cylindromatosis^62^ and Hao–Fountain syndrome^63^, respectively. In addition, other DUBs, including USP11 and USP20, have been implicated in tumorigenesis and are being explored as potential therapeutic targets^34,64^. The found peptide ligands could thus potentially be explored for specifically targeting the interactions of the binding DUBs. Related to disease associations, we note that only a small number of the identified SLiMs that bind ADs of USPs are directly affected by pathogenic mutations targeting key motif residues (Supp. Table 2). Specifically, we identified only three such mutations: a pathogenic ELOVL4 W246G variant affecting the putative USP11-binding site, and two pathogenic MYH11 variants (R1275L and R1275Q) that disrupt the putative USP20 UBPIM. However, in the case of ELOVL4 W246G, the affected region is located within an ER luminal domain, rendering a direct interaction with cytosolic USPs unlikely. Moreover, the MYH11 R1275L/Q variants are reported to exert their pathogenic effects primarily by disrupting myosin filament assembly, rather than by altering protein stability or regulatory interactions^65^. Taken together, these observations suggest that SLiMs engaging ADs of DUBs are rarely targeted directly by disease-associated mutations, in comparison to degrons, which appear to be more frequently perturbed in human disease^66,67^.

In summary, our study uncovered hitherto unknown peptide-binding functions for several DUBs and broadens the motif-based interactome of the DUBs. The results suggest intricate recognition mechanisms which may enhance the specificity and complex assembly of these enzymes. From a more applied perspective, there is growing interest in developing DUBTAC approaches to induce targeted protein stabilization. However, a major challenge in this field is targeting DUBs to substrates such that the DUB is optimally positioned for efficient deubiquitination. The reported DUB-binding peptides present a potentially attractive strategy to enable DUB-target proximity by utilizing the ADs and thereby enhance selectivity while avoiding off-target effects caused by targeting via the catalytic domains.

## Method

### Protein expression and purification

Proteins were expressed in *Escherichia coli (E.coli)* BL21-Gold (DE3) bacteria. The cDNAs encoding N-terminal 6-histidine-glutathione S-transferase tagged (6xHis-GST), 6xHis-tagged or GST-tagged protein domains in pETM33, pETM11, pGEX6P1 expression vectors respectively, were obtained from Genescript or kindly provided by MRC-PPU (Suppl. Data 1) The bacteria were transformed with the plasmid and grew at 37 °C in 2YT (5 g/L NaCl, 16 g/L tryptone and 10 g/L yeast extract) supplemented with Kanamycin (30 μg/mL) until reaching an OD_600_ of 0.7. Protein expression was induced with 0.3 mM isopropyl β-D-1 thiogalactopyranoside (IPTG) for 18 h at 18 °C while shaking. For zinc-finger domains, 50 μM of ZnSO_4_ was added during expression. The bacterial cultures were pelleted for 15 minutes at 4000 x g at 4 °C and stored at −20 °C until purification. The pellets were resuspended in lysis buffer (1× phosphate-buffered saline (PBS), 0.5 % Triton-X, 5 mM MgCl2, 10 μg/mL lysozyme, cOmplete^TM^ EDTA-free protease inhibitor cocktail (Roche), 10 μg/mL DNAseI) and incubated for 1 h shaking at 4 °C. The suspension was sonicated prior to phage selections or lysed using a cell disruptor (Constant Systems) prior to affinity measurements and peptide SPOT arrays, followed by centrifugation at 16,000 x g for 1 h at 4 °C. The supernatant was incubated with glutathione (GSH) Sepharose (Cytiva) or Ni2+ Sepharose (Cytiva) for 1 h at 4 °C while shaking. The beads with the bound protein were washed with 1x PBS and eluted with 10 mM reduced glutathione in 50 mM Tris pH 8.0. For fluorescence polarization experiments, the proteins were not eluted from the beads, and the 6xHis-GST tag was cleaved overnight on GSH Sepharose beads at 4 °C while shaking with PreScission Protease in cleavage buffer (50 mM Tris pH 7.5, 150 mM NaCl, 1 mM DTT). The sample containing the cleaved domain was separated from the tag and protease and buffer-exchanged to 50 mM sodium phosphate buffer pH 7.4 with 1 mM DTT using a PD-10 desalting column (Cytiva). The purity and size of the proteins were analyzed by SDS-PAGE electrophoresis.

### ProP-PD selections

The human disorderome library was used for four rounds of phage display selections^16^. GST-tagged, 6xHis-tagged or GST-6xHis purified proteins were used as baits and GST was used as control by immobilization on a 96-well MaxiSorp plate (Nunc) overnight at 4 °C while shaking (10 μg of protein in 100 μL PBS). The wells containing the immobilized proteins were blocked with 0.5% BSA in PBS for 1 hour at 4 °C while shaking. The phage library was prepared by precipitation in 1/5th volume PEG/NaCl (20% PEG-8000 + 0.4 M NaCl), incubated for 10 min on ice, followed by centrifugation at 10,000 × g for 10 min at 4 °C and resuspended in PBS. The GST-coated wells were washed four times with 0.05% Tween 20 in PBS, and 100 μL/well phage library was added and incubated 1 h at 4 °C while shaking. The protein-coated wells were washed four times with 0.05% Tween 20 in PBS, and the phage solution from the GST-coated wells was transferred to the protein-coated wells and incubated 2 h at 4 °C while shaking. The unbound phages were removed by washing 5 times with 0.05% Tween 20 in PBS, and the bound phages were eluted with 100 μL/well log-phase *E.coli* OmniMAX by incubating for 30 min at 37 °C while shaking. M13KO7 helper phages (10^11^ PFU/mL) were added to each well and incubated for 45 min at 37 °C while shaking. The hyper-infected bacterial cultures were transferred to 1 mL 2YT supplemented with carbenicillin (100 μg/mL), kanamycin (30μg/mL) and 0.3 mM IPTG and grown overnight at 37 °C while shaking. The bacterial cultures were pelleted by centrifugation at 2000 xg for 10 min at 4 °C. The pH of the phage supernatant was adjusted with 1/10th volume of 10× PBS, and any remaining bacteria were inactivated by heating at 65 °C for 10 min. The phage pools from each round were used for the next round of selections, and the process was repeated four times. To evaluate the enrichment of each round of selection, phage pool ELISA was performed. As before, 10 μg of GST and protein were immobilized on 96-well MaxiSorp plate (Nunc) overnight at 4 °C. The wells were blocked with 0.5% BSA in PBS for 1 h at 4 °C while shaking. The phage pools from each round were added to the respective GST-coated and protein-coated wells and incubated for 1 hour at 4 °C while shaking. The wells were washed four times with 0.05% Tween 20 in PBS, and 100 μL (1:5000) anti-M13 HRP-conjugated antibody (Nordic Biosite) was added to each well and incubated for 1 hour at 4 °C while shaking. The wells were washed four times with 0.05% Tween 20 in PBS and 1 time with PBS, and 100 μL TMB substrate was added to each well until a cyan color was developed. The reaction was stopped by addition of 100 μL 0.6 M H_2_SO_4_ to each well. The absorbance was measured for both GST-coated and protein-coated wells at 450 nm using the SpectraMax iD5 Multi-Mode Microplate Reader (Molecular Devices), and the binding enrichment was evaluated. The peptide-coding regions of binding-enriched phage pools were PCR-amplified and barcoded with Phusion High-Fidelity polymerase (Thermo Scientific). The PCR products were normalized with Mag-bind Total Pure NGS and cleaned up from 2% agarose gel using the QIAquick Gel extraction Kit (Qiagen). The samples were analyzed using the Illumina MiSeq platform.

### Peptide SPOT arrays

Peptides were synthesized on cellulose membranes with standard Fluoroenylmethylox-ycarbonyl (Fmoc) chemistry using a Multipep automated synthesizer at INTAVIS (Tübingen, Germany) or ordered from JPT (PepSpots). The membranes were activated with methanol for 5 min and washed three times for 5 min with TBST (50 mM Tris, 137 mM NaCl, 2.7 mM KCl, pH 8.0, 0.05% Tween-20). The membranes were incubated with blocking buffer (5% skim milk powder in TBST) for 2 h at room temperature while shaking. The membranes were incubated with the GST-tagged protein diluted at the desired concentration in blocking buffer and incubated overnight at 4 °C while shaking. The membrane was rinsed 3 times with TBST and incubated with HRP-conjugated anti-GST antibody (Cytiva, RPN1236; 1:3000 dilution) in blocking buffer for 1 h at 4 °C while shaking. The membrane was rinsed three times with TBST, and chemiluminescence detection was performed using ECL reagent (Clarity Max Western ECL substrate, 1705062, Bio-Rad) and ChemiDoc Imaging system (Bio-Rad). The images were analyzed in Fiji (ImageJ2 version 2.9.0).

### Site-directed mutagenesis

The zf-UBP domain mutations (USP20 zf-UBP: E55K-D59R, USP33 zf-UBP: E86K-D90R) were introduced by 2-step PCR with Pfu DNA polymerase (Agilent). After each step, the PCR product was digested with DpnI restriction enzyme (Thermo Scientific) and transformed into *E. coli*. The DNA was extracted from bacteria with QIAprep Spin Miniprep Kit (Qiagen) and the mutated sequences were confirmed by Sanger sequencing. Mutagenesis of mammalian USP20 and USP33 constructs was performed and obtained by Genescript, and for mammalian HIF1A, CEP192, and APBA2 constructs was performed and obtained by MRC PPU Reagents and Services.

### Fluorescence polarization

Affinity measurements were carried out using the SpectraMax iD5 Multi-Mode Microplate Reader (Molecular Devices) at 485 nm excitation and 535 nm emission. The measurements were performed at room temperature in a non-binding black half area 96-well plate (Corning) with a total volume of 50 μL per well. Peptides were obtained from GeneCust (France) with >95% purity. FITC-labeled peptides (N-terminally FITC-labeled without spacer and C-terminally amidated) were dissolved in dimethyl sulfoxide (DMSO), and unlabeled peptides (N-terminally acetylated and C-terminally amidated) were dissolved in 50 mM phosphate buffer pH 7.4. Saturation measurements were performed using a serial dilution of the protein domains in phosphate buffer (25 μL) and the addition of 10 nM FITC-labeled peptide (in phosphate buffer supplemented with 2 mM DTT) (25 μL). Displacement experiments were performed using a serial dilution of the unlabeled peptide in phosphate buffer (25 μL) and the addition of a master mix (25 μL) containing the protein (at a concentration of two-fold the K_D_ of the FITC-labeled peptide) in complex with 10 nM FITC-labeled peptide. All measurements were performed in three technical replicates. The data were analyzed using GraphPad Prism version 9.2.0 for MacOS (GraphPad Software, San Diego, California USA, www.graphpad.com). The saturation curves were fitted to the quadratic equation, and the displacement curves were fitted to a sigmoidal dose-response equation as described previously^68^.

### Computational modeling

Domain-motif interactions (Supplemental Table 8; Supplementary Fig. 7) were modeled using a local installation of AlphaFold3^18^, with 20 seeds per complex and with early stopping enabled when actifpTM ≥ 0.85. Model quality was also assessed and ranked using the actifpTM confidence metric, since flanking regions surrounding the motif can influence confidence score^60^. Structures were visualized using PyMOL^69^.

### GST-Pull-down assay

HEK293T cells were cultured in Dulbecco’s modified Eagle medium, high glucose, GlutaMAX™ Supplement (Gibco) supplemented with 10% fetal bovine serum (Gibco) and 1% penicillin/streptomycin at 37% and 5% CO2. The cells were transiently transfected with ATXN7L1 wild-type and mutant plasmids using jetOPTIMUS® transfection reagent (PolyPlus) following the manufacturer’s instructions; the medium was changed after 16 h and the cells were harvested after 48 h with trypsinization. The cells were lysed for 1 h rotating at 4 °C with lysis buffer (50 mM NaCl, 50 mM Tris, 1 mM EDTA pH 7.4, 0.1% NP-40 (Igepal), 1 mM DTT, cOmpleteTM EDTA-free protease inhibitor cocktail (Roche), PhosSTOP™ phosphatase inhibitors (Roche), 10 μg/mL DNAseI and RNAse). The samples were centrifuged for 1 h at 16,000 x g, and 20 μL of supernatant was added to 1-20 μL of GST- or GST-protein-bound glutathione beads purified as described above. The samples were incubated overnight rotating at 4 °C. The supernatant was removed, and the samples were washed three times with 1 mL of wash buffer (150 mM NaCl, 50 mM Tris pH 7.4, 0.05 % NP-40 (Igepal), 5% glycerol, 1 mM DTT). Samples from input and pull-downs were mixed with 4x Laemmli Loading dye (Bio-rad) or 2x loading dye (NuPAGE), boiled at 95 °C for 5 min, and stored at −20 °C. Western blots were performed after running the samples in SDS-PAGE gels with Kaleidoscope (Bio-rad) or PageRuler™ Prestained Protein Ladder (Thermo Scientific) and transferred to a nitrocellulose membrane using the Trans-Blot Turbo transfer system (Bio-rad). The membranes were incubated in blocking buffer (5% skim milk in PBST (1x PBS and 0.1% Tween-20). For immunoblotting, the membranes were incubated with 1:5000 primary mouse anti-FLAG® M2 antibody (Sigma Aldrich) overnight and 1:5000 rabbit anti-GST Tag antibody (Sigma Aldrich) for 1 h in 2.5% skim milk in PBST. The membranes were washed 3 times for 5 min with PBST. For visualization, the membranes were incubated with 1:5000 secondary antibody Goat anti-mouse IRDye® 680RD or Goat anti-rabbit IRDye® 800CW (LI-COR) accordingly in 5% skim milk in PBST for 1 h at room temperature. The membranes were washed three times for 5 min with PBST and visualized by scanning at 700 nm and 800 nm with Odyssey XF (LI-COR) imaging system.

### Transient transfection for IP and IP-MS experiments

For transfection, 1x10^6^ HEK293 cells were seeded in 10 cm dishes with DMEM, 10% FBS, 1% L-G (without antibiotic) medium, incubated at 37 °C, with 5 % CO_2_ for 24 h. Cells were transfected with a transfection mix containing 970 mL of Opti-MEM (Thermo Fisher Scientific), 20 μL of PEI stock (1 mg/mL) and 5 mg of the desired cDNA. For co-transfection, 2.5 μg of DNA of each plasmid was used. The mix was vortexed for 15 s and incubated at room temperature for 15–20 min. The transfection mix was added to the dish and gently mixed. Plates were incubated at 37 °C for 36–48 h before harvesting. For USP33 constructs, 5 μM Bortezomib (proteasome inhibitor) was added 4 hours before harvesting the cells. To detect endogenous and transfected HIF1A, 100 μM of CoCl_2_ was added to cells overnight to mimic hypoxia, and 5 μM Bortezomib was added 4 hours before the cells were harvested.

### Immunoprecipitation of FLAG fusion proteins

FLAG fusion proteins were immunoprecipitated from HEK293 cells transfected with plasmids encoding the protein of interest using the anti-FLAG M2 affinity gel from Sigma-Aldrich. Cell pellets were lysed with 100–200 μL/per dish lysis buffer (Pierce RIPA buffer, 1X protease inhibitor (Roche), 50 unit/μL of Benzonase Nuclease) rotating at 4 °C for 30–45 min. 20 μL of anti-FLAG M2 affinity gel slurry per plate was equilibrated with IP buffer (10 mM Tris pH 7.5, 137 mM NaCl, 0.1% NP-40). The cell lysate was clarified by centrifugation at 17,000 x g, at 4 °C for 12 min, and the supernatant was transferred to a clean, pre-chilled low-binding Eppendorf tube. The protein concentration was estimated with a Bradford assay, and an equal amount of protein per IP (0.5–2 mg) diluted with 500–750 μL of IP buffer was loaded into an Eppendorf tube with equilibrated anti-FLAG M2 affinity gel and incubated in the rotator in the cold room for 2 h. The IP samples were centrifuged at 6500 x g at 4 °C for 30 s. The supernatant containing the unbound proteins was transferred to a clean tube. The resin was washed three times with 500 μL of TBS, centrifuged at 6500 x g at 4 °C for 30 s. The bound FLAG fusion protein was eluted with 100 μL of 150 ng/μL 3X FLAG peptide (Sigma) in TBS and incubated in the cold room for 1 h or overnight. The tube was centrifuged at 6500 x g at 4 °C for 30 s; the eluted proteins were transferred to a clean tube. The eluted proteins were prepared for analysis by western blot or Mass Spectrometry.

### Co-Immunoprecipitation of HA and FLAG fusion proteins

HA-tagged protein was immunoprecipitated from HEK293 cells transfected with plasmids encoding the protein of interest using Pierce anti-HA magnetic beads (Thermo Scientific). Cells were lysed with 100-200 μL of lysis buffer (Pierce RIPA buffer, 1X protease inhibitor (Roche), 50 unit/mL of Benzonase Nuclease), rotating at 4 °C for 30–45 min. 20 μL of magnetic beads slurry per 10 cm plate was equilibrated with IP buffer on a magnetic stand. The cell lysate was centrifuged at 17,000 x g at 4 °C for 12 min. The supernatant was transferred to a clean, pre-chilled, low-binding Eppendorf tube. The protein concentration was estimated with a Bradford assay; 1–2 mg of protein diluted with 300 μL of TBS was loaded on equilibrated magnetic beads and incubated on the rotator at 4 °C for 2 h. The IP tube was placed on the magnetic stand, and the supernatant was removed. The unbound proteins were transferred to a clean tube. The magnetic beads were washed two times with 300 µL IP buffer and one time with TBS on the magnetic stand. The bound HA-tagged protein was eluted with 100 µL of 2X LDS without reducing agent and incubated at 70 °C for 5 min. The tube was placed on the magnetic stand, and the eluted proteins were transferred to a fresh tube. 10 μL of NuPAGE™ Sample Reducing Agent (10X) was added to the samples and stored at −20 °C for further analysis by Western blot. 10 μg of protein and 15 µL of IP were loaded for input and IP samples for Western blotting.

### Western blotting

Western blot samples were resolved on an SDS-PAGE gel using MES buffer for proteins of ≤ 50 kDa, and MOPS for proteins of ≥ 50 kDa. The gel was rinsed with distilled water and transferred onto a nitrocellulose membrane in 1X transfer buffer (Tris base 3.0 g/L, Glycine 14.4 g/L, 20% Methanol) at 100 V for 30–35 min in the cold room, using the Criterion Western Protein Blotter Transfer System (BioRad). After the transfer, Ponceau staining was performed to check the efficiency of the transfer. The membrane was washed with MilliQ water to remove the Ponceau stain and then incubated in the blocking buffer (5% BSA in 1X TBST) at room temperature for 1 hour, with shaking. The blocking solution was removed and then incubated with the corresponding primary antibody overnight with shaking at 4 °C. The next day, the primary antibody was removed, and the membrane was washed with TBST three times at room temperature with shaking for 10 min. Next, the membrane was incubated with a secondary antibody diluted 1:5000 in the blocking buffer for 1 h. The membrane was washed with TBS three times at room temperature, shaking for 10 min. After washing, the membrane was developed with Clarity Western ECL Substrate (BioRad) using the ChemiDoc Imaging System (BioRad).

### IP-Mass Spectrometry

Immunoprecipitation (IP) was carried out as described above; four technical replicates of each IP condition were performed, and the empty vector was used as a negative control. The eluted protein was processed following the Strap-micro protocol (Protifi). The IP elution containing 1–100 μg protein was 1:1 mixed with 2X lysis buffer (10% SDS, 100 mM TEAB pH 8.5). Subsequently, the sample was reduced, alkylated, and denatured with 5 mM TCEP at 55 °C for 15 min, 400 mM chloroacetamide at room temperature for 10 minutes, and ~2.5% phosphoric acid, respectively. Samples were vortexed, mixed with 165 μL of 100 mM TEAB in 90% methanol, and passed through an S-Trap micro column at 4000 g for 30 s to trap the proteins. The column was washed with 150 μL of 100 mM TEAB in 90% methanol (at 4,000 x g for 30 seconds) three times. The S-Trap column was centrifuged again and transferred to a clean 2 mL tube for digestion with 20 μL of digestion buffer (1 μg trypsin per 10 μg sample in 50 mM TEAB), at 37 °C overnight in a thermomixer without shaking. The digested peptides were eluted with 40 mL of 50 mM TEAB in water, 40 mL of 0.2 % formic acid in water, and 40 mL of 50 % acetonitrile in water, centrifuging at 4000 x g for 1 min each time. The three elutions were pooled in a low-binding tube, placed on dry ice until frozen, dried in a SpeedVac vacuum (Thermo Scientific), and proceeded with the LCMS analysis.

### LC-MS analysis

Eluted peptides were resuspended in water supplemented with 0.015% dodecyl maltoside and 0.1% formic acid and were injected on a Vanquish Neo UHPLC System operating in trap and elute mode coupled to an Orbitrap Astral Mass Spectrometer (Thermo Fisher Scientific). Peptides were loaded onto a PepMap Neo Trap Cartridge (Thermo Fisher Scientific #174500) and analyzed on a C18 EASY-Spray HPLC Column (Thermo Fisher Scientific #ES906) with a 11.8 minute gradient from 1% to 55% Buffer B (Buffer A: 0.1% formic acid in water; Buffer B: 0.08% formic acid in 80:20 acetonitrile: water, 0.7 min at 1.8 µL/min from 1% to 4% B, 0.3 min at 1.8 µL/min from 4% to 8% B, 6.7 min at 1.8 µL/min from 8 to 22.5% B, 3.7 min at 1.8 µL/min from 22.5 to 35% B, 0.4 min at 2.5 µL/min from 35 to 55% B). Eluted peptides were analyzed using data-independent acquisition mode on the mass spectrometer.

### Data analysis

Peptides were searched against the Uniprot Swissprot Human database (released on 02/01/2023) supplemented with the USP20 and USP33 mutants using DiaNN (v1.9.0)^62^ operating in library-free mode. Data filtering and statistical analysis were carried out in Python (v3.9.0) using the packages pandas, numpy, sklearn, scipy, rpy2, Plotnine, and Plotly and R (v4.4.3) using the package limma.

Protein groups identified with a single peptide or quantified in fewer than three replicates in at least 1 condition were filtered out. Protein group intensities were median normalized and missing values were imputed using a Gaussian distribution centered on the median with a downshift of 1.8 and width of 0.3 (relative to the standard deviation). Protein regulation was then assessed using LIMMA eBayes, and P-values were adjusted using Benjamini-Hochberg multiple hypothesis correction. Proteins were considered significantly regulated if their corrected P-value was smaller than 0.05 and their fold change was greater than 1.5 or smaller than 1/1.5. Following statistical analysis, differential analysis visualization and Data annotation were performed with CURTAIN. Volcano plots were generated by plotting the log2 fold change (x-axis) against the -log10 p-value (y-axis) of differential expression, setting significance thresholds of p-value of <0.05 and a fold change cutoff of 2 (or log2 (2)). Plots were exported to Illustrator for figure preparation.

### Reporting summary

Further information on research design is available in the Nature Portfolio Reporting Summary linked to this article.

## Supplementary information

Supplementary Information

Description of Additional Supplementary Files

Supplementary Data 1

Supplementary Data 2

Supplementary Data 3

Supplementary Data 4

Supplementary Data 5

Supplementary Data 6

Supplementary Data 7

Supplementary Data 8

Reporting Summary

Transparent Peer Review file

## Source data

Source Data

## Data Availability

The protein interaction data generated in this study has been deposited to the IMEx (http://www.imexconsortium.org) consortium through the IntAct^70^ database under the accession code IM-30531 https://www.ebi.ac.uk/intact/imex/main.xhtml;jsessionid=2D542E0FB613EFEB8C1D67C7A89E5951?conversationContext=1. MS data has been submitted to the PRIDE portal (Project accession: PXD068553 https://www.ebi.ac.uk/pride/archive/projects/PXD068553). ProP-PD data is available in the ProP-PD portal https://slim-tools.org/proppd/. Data is also available in the Supplementary tables (ProP-PD data: Supplementary Table 2; MS data: Supplementary Table 6). Source data are provided with this paper.
