# Supplementary material for: Systematic discovery of motif-based interactions of the auxiliary domains of USP family deubiquitinases
Source: Nat Commun. 2026 May 18;17:6531. doi: 10.1038/s41467-026-73047-7 (PMC13376213; doi:10.1038/s41467-026-73047-7)
Supplement: Supplementary file 1 — Supplementary Information [file 41467_2026_73047_MOESM1_ESM.pdf]

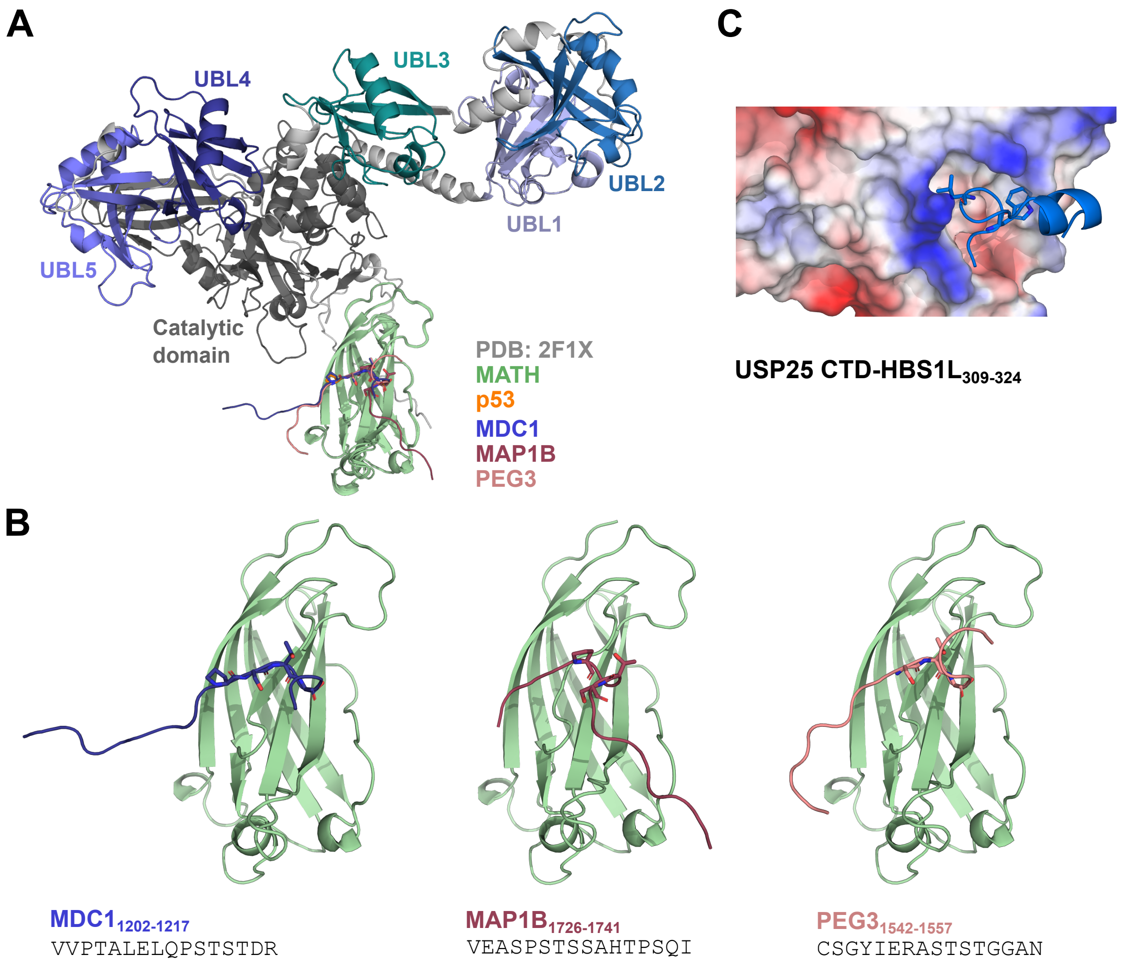

Supplementary Figure 1. Peptides binding to the USP7 MATH and USP25 CTD domain. A. Crystal structure of MATH domain of USP7 bound to a p53 peptide (PDB: 2F1X) superimposed with AF3 models of the USP7 full-length predicted to bind to peptides identified in ProP-PD selections. **B.** AF3 models of the MATH domain of USP7 bound to right: MDC1$_{1202\text{-}1217}$ (ipTM: 0.77), middle: MAP1B$_{1726\text{-}1741}$ (ipTM: 0.82) and left: PEG3$_{1542\text{-}1557}$ peptides (ipTM: 0.83). The motif key residues are shown in stick representation (see Figure 1D). **C.** AF3 model of the USP25 CTD in complex with the HBS1L$_{309\text{-}324}$ peptide (ipTM: 0.6) with CTD domain shown with surface electrostatic potential representation.

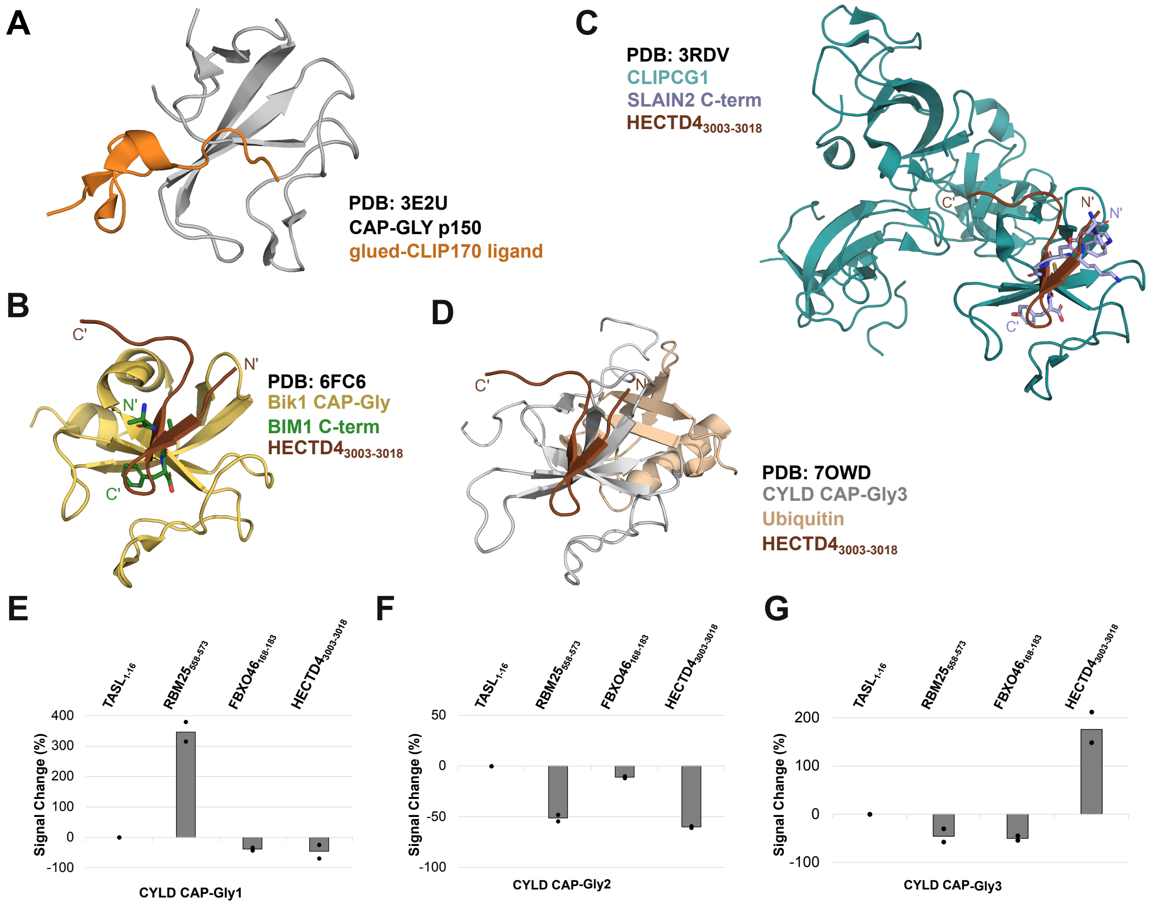

Supplementary Figure 2. Peptide binding of CAP-Gly domains. A. Crystal structure of the CLIP170 peptide (orange) in complex with the CAP-Gly domain of p150 glued (PDB: 3E2U). **B.** Crystal structure of Bik1 CAP-Gly binding to BIM1 C-terminal peptide (green) (PDB: 6FC6) superimposed with the AF3 model of HECTD4$_{3003-3018}$ peptide (brown) predicted to bind to the CAP-Gly3 domain of CYLD. **C.** Crystal structure of CLIPCG1 CAP-Gly binding to SLAIN2 C-terminal peptide (purple) (PDB: 3RDV) superimposed with the AF3 model of HECTD4$_{3003-3018}$ peptide (brown) predicted to bind to the CAP-Gly3 domain of CYLD. **D.** Crystal structure of CYLD CAP-Gly3 binding to ubiquitin (nude) (PDB: 7OWD) superimposed with the AF3 model of HECTD4$_{3003-3018}$ peptide (brown). **E**. CYLD CAP-Gly1 SPOT array signal intensity change (in percentage) of the wild-type TASL$_{1-16}$ peptide compared to RBM25$_{558-573}$, FBXO46$_{168-183}$ and HECTD4$_{3003-3018}$ peptides. **F.** CYLD CAP-Gly2 SPOT array signal intensity change (in percentage) of the wild-type TASL$_{1-16}$ peptide compared to RBM25$_{558-573}$, FBXO46$_{168-183}$ and HECTD4$_{3003-3018}$ peptides. **G.** CYLD CAP-Gly1 SPOT array signal intensity change (in percentage) of the wild-type TASL$_{1-16}$ peptide compared to RBM25$_{558-573}$, FBXO46$_{168-183}$ and HECTD4$_{3003-3018}$ peptides. E-G. Signal intensities were normalized to the wild-type TASL$_{1-16}$ peptide (individual points) and presented as average percent signal difference (bar).

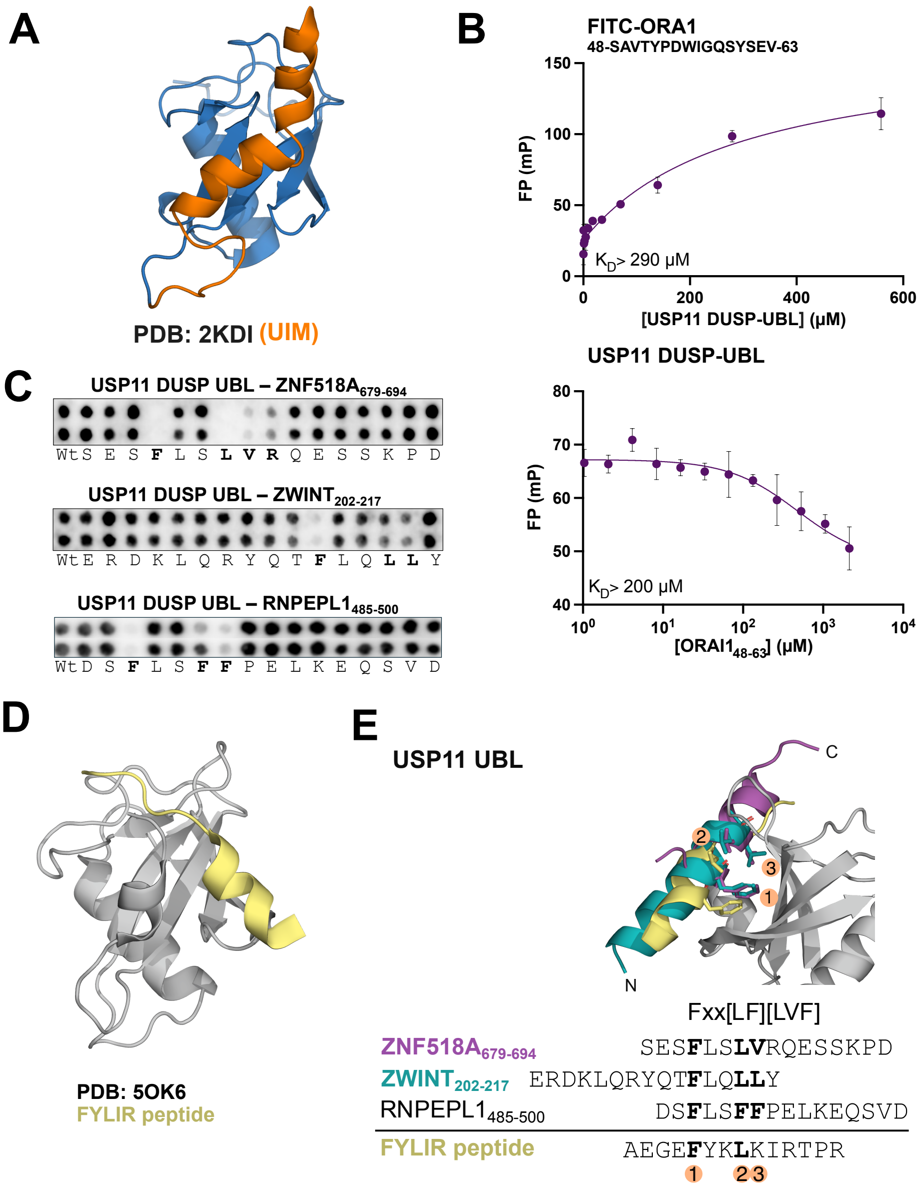

Supplementary Figure 3. Peptide binding of the USP19 UBL and the USP11 DUSP UBL domains. A. NMR structure of the UIM motif binding to ubiquitin (PDB: 2KDI). **B**. FP-monitored affinity measurements of USP11 DUSP-UBL domain with labeled (direct binding) and unlabeled (competitive binding) ORAI1 peptide. Measurements were performed in technical triplicates, and the data are presented as means ± SD. **D.** Crystal structure of the USP11 UBL domain binding to the FYLIR peptide (PDB: 5OK6). **E.** AF3 models of the $ZNF518A_{679\text{-}694}$ and $ZWINT_{202\text{-}217}$ peptides superimposed with the solved structure of USP11 DUSP UBL-FYLIR complex. The Fxx[LF][LVF] motif residues are indicated and shown as sticks.

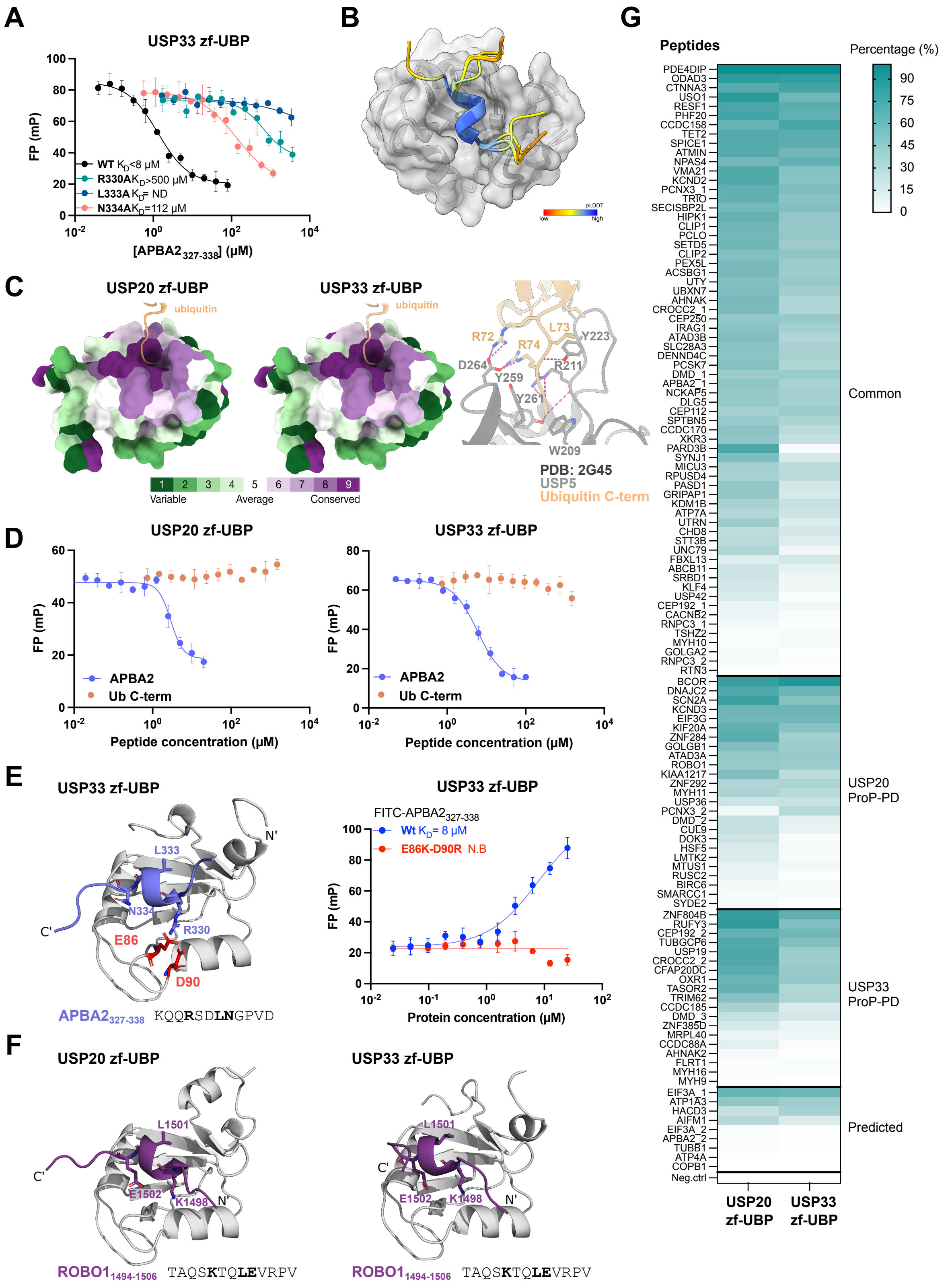

Supplementary Figure 4. USP20 and USP33 zf-UBP domain peptide binding analysis. A. Competitive FP-monitored affinity measurements of USP33 zf-UBP binding to wild-type and mutant APBA2$_{327-338}$ peptides. Measurements were performed in technical triplicates, and the data are presented as means ± SD. ND: not determined. **B.** Superimposition of the AF3 models of the APBA2 and ROBO1 peptides in complex with USP20 and USP33 zf-UBP domains. The peptides were modeled with high confidence at the peptide region (pLDDT> 80 over the motif residues). **C.** Left, middle: Conservation of the zf-UBP domain and ubiquitin binding pocket of USP20 and USP33 zf-UBP domains. Conservation among organisms is demonstrated based on ConSurf[1] (Yariv

et al, 2023) Right: USP5 zf-UBP domain (grey) binding to the C-terminal tail of ubiquitin (nude) (PDB: 2G45). **D.** FP-monitored affinity measurements of the ubiquitin C-terminal peptide with USP20 and USP33 zf-UBP domains. The APBA2 peptide affinity measurements are shown for comparison (see Figure. 4H-J). Measurements were performed in technical triplicates, and the data are presented as means ± SD. **E.** Left: AF3 model of the interactions between the $APBA2_{327\text{-}338}$ peptide and the zf-UBP domain of USP33 (ipTM:0.83; pLDDT> 80 over the motif residues). Right: FP-monitored affinity measurements between wild-type and double mutant zf-UBP domains of USP33 and the $APBA2_{327\text{-}338}$ peptide. Measurements were performed in technical triplicates, and the data are presented as means ± SD. **F.** AF3 models of the ROBO1 peptide with the zf-UBP domains (USP20 ipTM: 0.7, USP33 ipTM: 0.66);pLDDT> 80 over the motif residues). **G.** Heatmap representation of peptide SPOT array analysis of 120 distinct peptides binding to USP20 and USP33 zf-UBP domains. Peptides were found in phage selections against both USP20 and USP33 ("Common"), in either of the two USP20 and USP33 zf-UBP domain datasets or were predicted to contain the motif. The background signal was subtracted, and the signal intensities of the peptides were normalized to the highest intensity. Peptide sequences and SPOT array results are available in the Suppl. Table 4.

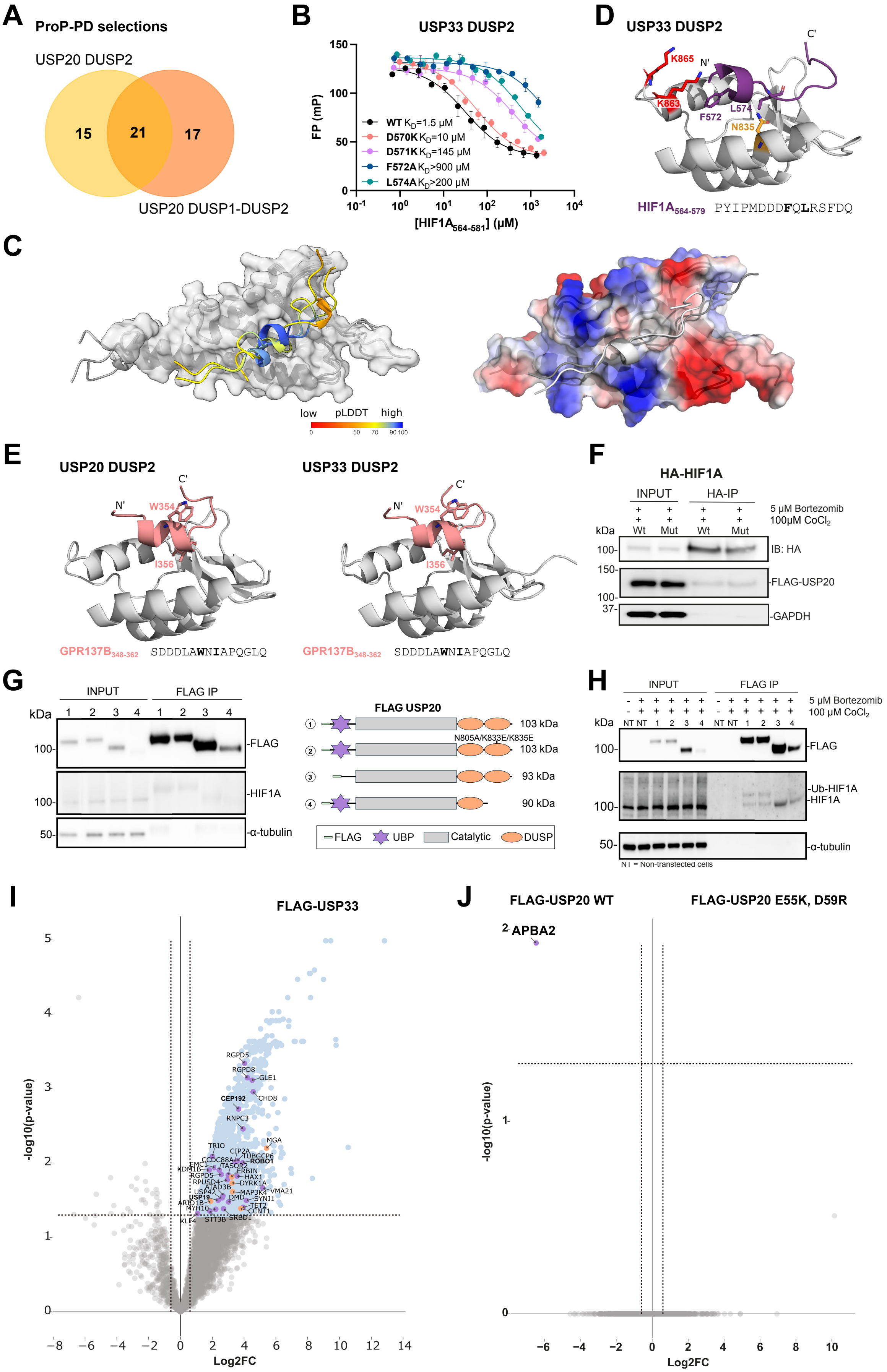
A
ProP-PD selections
USP20 DUSP2
15
21
17
USP20 DUSP1-DUSP2
B
USP33 DUSP2
FP (mP)
WT K_D=1.5 μM
D570K K_D=10 μM
D571K K_D=145 μM
F572A K_D>900 μM
L574A K_D>200 μM
[HIF1A564-581] (μM)
D
USP33 DUSP2
K865
K863
F572
L574
N835
HIF1A564-579 PYIPMDDDFQLRSFDQ
C
low pLDDT high
E
USP20 DUSP2
USP33 DUSP2
W354
I356
GPR137B348-362 SDDDLAWNIAPQGLQ
GPR137B348-362 SDDDLAWNIAPQGLQ
F
HA-HIF1A
INPUT
HA-IP
5 μM Bortezomib
100μM CoCl2
Wt Mut Wt Mut
IB: HA
FLAG-USP20
GAPDH
G
INPUT
FLAG IP
FLAG
HIF1A
α-tubulin
FLAG USP20
103 kDa
N805A/K833E/K835E
103 kDa
93 kDa
90 kDa
FLAG UBP Catalytic DUSP
H
INPUT
FLAG IP
5 μM Bortezomib
100 μM CoCl2
NT NT 1 2 3 4 NT NT 1 2 3 4
FLAG
Ub-HIF1A
HIF1A
α-tubulin
NT = Non-transfected cells
I
FLAG-USP33
-log10(p-value)
Log2FC
RGPD5
RGPD8
GLE1
CEP192
CHD8
RNPC3
MGA
TRIO
CIP2A
CCDC88A
TUBGCP6
ROBO1
ERBIN
HAX1
DYRK1A
MAP3K4
VMA21
USP19
SYNJ1
TET2
CCNT1
KLF4
STT3B
SRBD1
J
FLAG-USP20 WT
FLAG-USP20 E55K, D59R
APBA2
-log10(p-value)
Log2FC

Supplementary Figure 5. USP20 and USP33 DUSP2 domain peptide binding analysis and full-length interaction profiling. A. Venn diagram illustrating the overlap of peptide-binding regions identified as ligands for the USP20 DUSP2 and tandem DUSP1-DUSP2 domains through ProP-PD selections. **B.** Competitive FP-monitored affinity measurements of USP33 DUSP2 binding to wild-type and mutant HIF1A$_{564-579}$ peptides. Measurements were performed in technical triplicates, and the data are presented as means ± SD. **C.** Left: Superimposition of the AF3 models of the HIF1A and GPR137B peptides in complex with the DUSP2 domains. The peptides were modeled with high confidence at the [FW]x[IL] motif region. Right: AF3 model of HIF1A binding to USP20 DUSP2 in electrostatic surface potential representation. **D.** AF3 model of USP33 domain binding to HIF1A$_{564-579}$ (pLDDT>90 values over the residues; ipTM:0.7). **E.** AF3 models of the GPR137B peptide with the DUSP2 domains (USP20 ipTM:0.77, USP33 ipTM: 0.72; pLDDT> 90 over the motif residues). **F.** Co-immunoprecipitation experiments of FLAG-USP20 with wild-type and mutant HA-HIF1A (F572A-L574A) in HEK293 cells, repeated in at least biological duplicate experiments. **G.** Left: Immunoprecipitation of endogenous HIF1A with wild-type USP20 and predicted DUSP2 binding pocket mutants or truncated zf-UBP or DUSP2 domains in normoxia, repeated in at least biological duplicate experiments. Right: Schematic representation of the USP20 FLAG-tagged constructs. **H.** Immunoprecipitation of endogenous HIF1A with wild-type USP20 and predicted DUSP2 binding pocket mutants or truncated zf-UBP or DUSP2 domains in hypoxia mimic conditions (see panel 5G for descriptions of constructs used shown by numbers 1-4; repeated in at least biological duplicates). **I.** FLAG-USP33 IP-MS analysis compared to the empty pCMV vector in HEK293 cells. Indicated are the interactors also identified in ProP-PD experiments for zf-UBP (purple) and DUSP2 domain (orange). **J.** IP-MS analysis of the wild-type and predicted binding pocket mutant FLAG-USP20 (E55K/D59R). The APBA2 interaction with the binding pocket mutant is significantly affected. For more information on IP-MS data (I, J) see Suppl. Table 6.

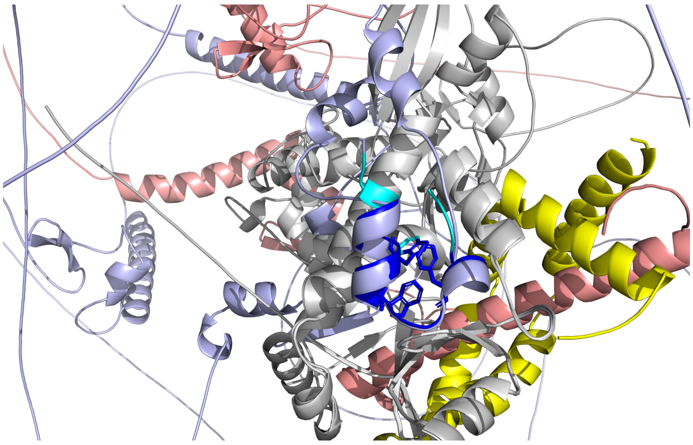

Supplementary Figure 6. AF3 model of the SAGA complex. Full-length ATXN7L1 (light blue), USP22 (grey), ENY2 (yellow) and ATXN7L3 (light red)), overlayed with complex of USP22 zf-UBL-ATXN7L1 (coloured by the confidence of the AF3 model).

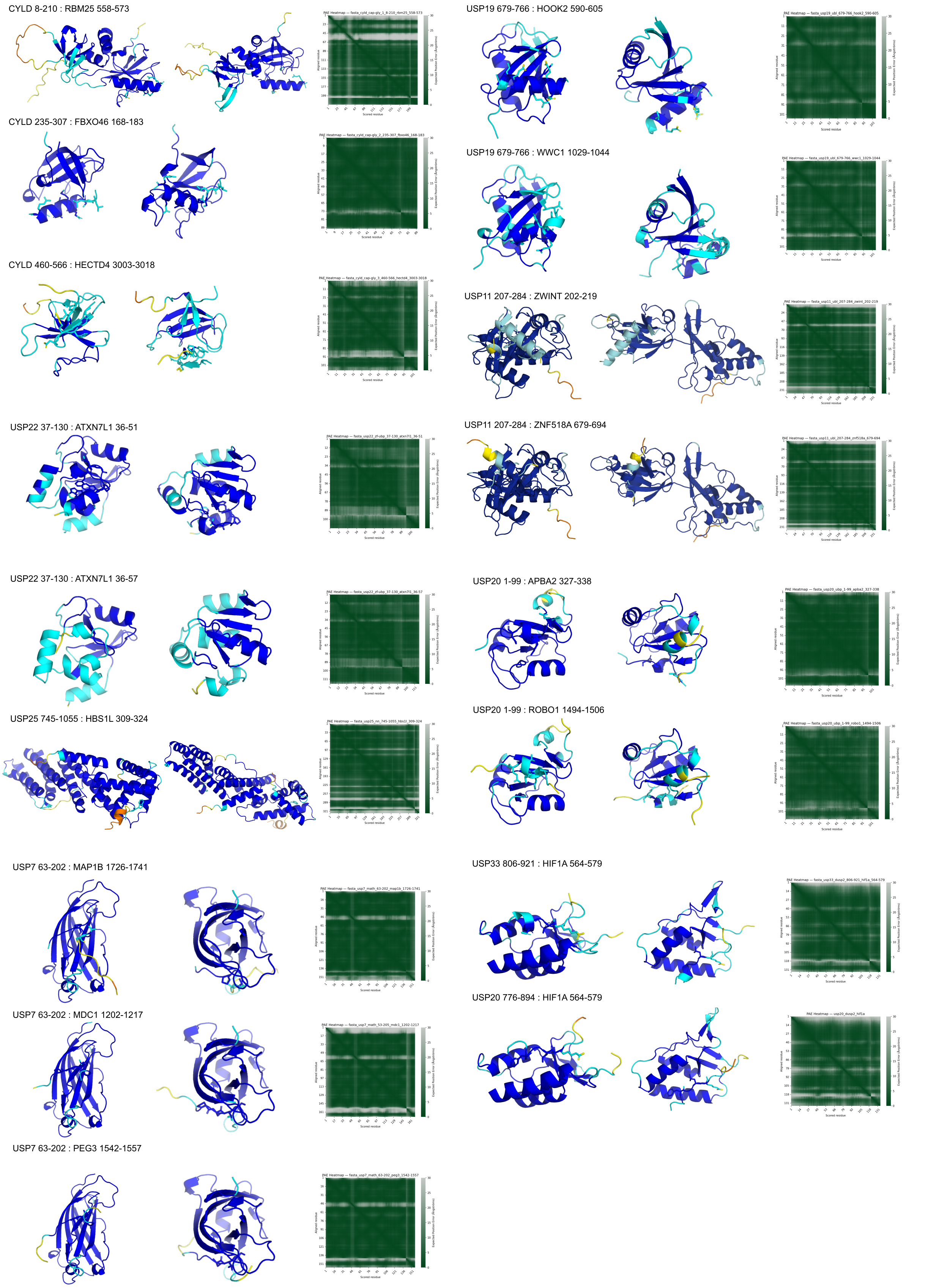

Supplementary Figure 7. Compiled AF models with pLDDT score overlaid from two viewing angles, together with the Predicted aligned error (PAE).

Supplementary References

1. Yariv, B., Yariv, E., Kessel, A., Masrati, G., Chorin, A. B., Martz, E., Mayrose, I., Pupko, T., and Ben-Tal, N. (2023) Using evolutionary data to make sense of macromolecules with a "face-lifted" ConSurf, *Protein Sci 32*, e4582.
