# Supplementary material for: Systematic discovery of motif-based interactions of the auxiliary domains of USP family deubiquitinases
Source: Nat Commun. 2026 May 18;17:6531. doi: 10.1038/s41467-026-73047-7 (PMC13376213; doi:10.1038/s41467-026-73047-7)
Supplement: Supplementary file 2 — Description of Additional Supplementary Files [file 41467_2026_73047_MOESM2_ESM.pdf]

Description of Additional Supplementary Files

File Name: Supplementary Data 1

Description: Collection of protein expression constructs used in this study

Tab S1A: List of all bait proteins used in ProP-PD selections, including information on amino acid sequence and plasmids.

Tab S1B: List of constructs of mutated zf-UBP and DUSP2 domains, including the amino acid sequence with the mutations.

Tab S1C: List of constructs used for mammalian expression.

File name: Supplementary Data 2

Description: ProP-PD results of bait proteins screened.

Tab S2A List of ProP-PD results for all the baits screened in the study. The peptide datasets were analysed and annotated with PepTools.

Tab S2B List of ProP-PD results with collapsed amino acid regions of overlapping peptides.

Tab S2C Data for USP7 MATH domain generated in previous studies.

File name: Supplementary Data 3

Description: List of all affinity data provided Overview of affinity measurements conducted for this study, including K_D values and peptides sequences.

File name: Supplementary Data 4

Description: USP20 and USP33 zf-UBP SPOT arrays. Peptide SPOT array to validate binding of peptides from ProP-PD experiments and peptides predicted to have the motif based on PSSM analysis. The peptides were divided into four categories (common: found in both ProP-PD datasets, USP20 or USP33: found only in one dataset and predicted. Peptides were synthesized in triplicates and the average signal intensity was calculated for each peptide. Signal intensities were normalized to the highest intensity value (set as 100%).

Tab S4A USP20 zf-UBP

Tab S4B USP33 zf-UBP

File name: Supplementary Data 5.

Description: USP20 and USP33 DUSP2 SPOT array. Peptide SPOT array to validate binding of peptides from ProP-PD experiments. The peptides were divided into three categories (common: found in both ProP-PD datasets and USP20 or USP33: found only in one dataset. Peptides were synthesized in triplicates and the average signal intensity was calculated for each peptide.

Signal intensities were normalized to the highest intensity value (set as 100%).

Tab S5A USP20 DUSP2
Tab S5B USP33 DUSP2

File name: Supplementary Data 6.

Description: Data analysis from IP-MS experiments performed in HEK293 cells under normoxic conditions (EV: Empty vector). Differential protein levels were assessed using the LIMMA framework (empirical Bayes moderated t-statistics; R package limma v4.4.3). All statistical tests were two-sided. Resulting P-values were adjusted for multiple comparisons using the Benjamini–Hochberg false discovery rate (FDR) correction. Proteins were considered significantly regulated if the adjusted P-value was <0.05 and the fold change exceeded 1.5 (or was <1/1.5).

Tab S6A FLAG-USP20 vs EV
Tab S6B FLAG-USP33 vs EV
Tab S6C FLAG-USP20 zf-UBP mutant vs EV
Tab S6D FLAG-USP33 zf-UBP mutant vs EV
Tab S6E FLAG-USP20-ΔUBP vs EV
Tab S6F FLAG-USP20-ΔDUSP2 vs EV
Tab S6G FLAG-USP33-ΔUBP vs EV
Tab S6H FLAG-USP33-ΔDUSP2 vs EV

File name: Supplementary Data 7.

Description: Predicted SLiMs among MS derived USP20/33 interactors. IP-MS data was analysed as described previously: Differential protein levels were assessed using the LIMMA framework (empirical Bayes moderated t-statistics; R package limma v4.4.3), usingl two-sided statistical tests. Resulting P-values were adjusted for multiple comparisons using the Benjamini–Hochberg false discovery rate (FDR) correction.

Tab S7a Overview of zf-UBP and DUSP2 binding motifs among putative USP20 and USP33 interactors identified by IP-MS.
Tab S7b Motif distribution in USP20 ligands.
Tab S7c Motif distribution in USP33 ligands.

File name: Supplementary Data 8.

Description: Sequence information used for AlphaFold modelling of complexes, together with ipTM and actifPTM scores.
