# Supplementary material for: Systematic discovery of motif-based interactions of the auxiliary domains of USP family deubiquitinases
Source: Nat Commun. 2026 May 18;17:6531. doi: 10.1038/s41467-026-73047-7 (PMC13376213; doi:10.1038/s41467-026-73047-7)
Supplement: Supplementary file 11 — Reporting Summary [file 41467_2026_73047_MOESM11_ESM.pdf]

nature portfolio

Corresponding author(s): Ylva Ivarsson

Last updated by author(s): Mar 30, 2026

Reporting Summary

Nature Portfolio wishes to improve the reproducibility of the work that we publish. This form provides structure for consistency and transparency in reporting. For further information on Nature Portfolio policies, see our Editorial Policies and the Editorial Policy Checklist.

Statistics

For all statistical analyses, confirm that the following items are present in the figure legend, table legend, main text, or Methods section.

n/a	Confirmed	
☐	☒	The exact sample size (*n*) for each experimental group/condition, given as a discrete number and unit of measurement
☐	☒	A statement on whether measurements were taken from distinct samples or whether the same sample was measured repeatedly
☐	☒	The statistical test(s) used AND whether they are one- or two-sided<br>*Only common tests should be described solely by name; describe more complex techniques in the Methods section.*
☒	☐	A description of all covariates tested
☒	☐	A description of any assumptions or corrections, such as tests of normality and adjustment for multiple comparisons
☐	☒	A full description of the statistical parameters including central tendency (e.g. means) or other basic estimates (e.g. regression coefficient) AND variation (e.g. standard deviation) or associated estimates of uncertainty (e.g. confidence intervals)
☐	☒	For null hypothesis testing, the test statistic (e.g. *F*, *t*, *r*) with confidence intervals, effect sizes, degrees of freedom and *P* value noted<br>*Give P values as exact values whenever suitable.*
☒	☐	For Bayesian analysis, information on the choice of priors and Markov chain Monte Carlo settings
☒	☐	For hierarchical and complex designs, identification of the appropriate level for tests and full reporting of outcomes
☒	☐	Estimates of effect sizes (e.g. Cohen's *d*, Pearson's *r*), indicating how they were calculated

Our web collection on statistics for biologists contains articles on many of the points above.

Software and code

Policy information about availability of computer code

Data collection	Uniprot Swissprot Human database (released on 02/01/2023)
Data analysis	We used GraphPad Prism version 9.2.0 for MacOS (GraphPad Software, San Diego, California USA, www.graphpad.com) to analyse affinity data. We also used DiaNN (v1.9.0)61, Python (v3.9.0) and R (v4.4.3) and CURTAIN (https://curtain.proteo.info).

For manuscripts utilizing custom algorithms or software that are central to the research but not yet described in published literature, software must be made available to editors and reviewers. We strongly encourage code deposition in a community repository (e.g. GitHub). See the Nature Portfolio guidelines for submitting code & software for further information.

Data

Policy information about availability of data

All manuscripts must include a data availability statement. This statement should provide the following information, where applicable:

- Accession codes, unique identifiers, or web links for publicly available datasets
- A description of any restrictions on data availability
- For clinical datasets or third party data, please ensure that the statement adheres to our policy

The protein interaction data generated in this study has been deposited to the IMEx (http://www.imexconsortium.org) consortium through the IntAct70 database under the accession code IM-30531 https://www.ebi.ac.uk/intact/imex/main.xhtml;jsessionid=2D542E0FB613EFEB8C1D67C7A89E5951?conversationContext=1. MS data has been submitted to the PRIDE portal (Project accession: PXD068553 https://www.ebi.ac.uk/pride/archive/projects/PXD068553). ProP-PD data is available in the ProP-PD portal https://slim-tools.org/proppd/. Data is also available in the Supplementary tables (ProP-PD data: Supplementary Table 2; MS data:

Supplementary Table 6). Source Data are provided with this paper.

Research involving human participants, their data, or biological material

Policy information about studies with human participants or human data. See also policy information about sex, gender (identity/presentation), and sexual orientation and race, ethnicity and racism.

Reporting on sex and gender	*Use the terms sex (biological attribute) and gender (shaped by social and cultural circumstances) carefully in order to avoid confusing both terms. Indicate if findings apply to only one sex or gender; describe whether sex and gender were considered in study design; whether sex and/or gender was determined based on self-reporting or assigned and methods used. Provide in the source data disaggregated sex and gender data, where this information has been collected, and if consent has been obtained for sharing of individual-level data; provide overall numbers in this Reporting Summary. Please state if this information has not been collected. Report sex- and gender-based analyses where performed, justify reasons for lack of sex- and gender-based analysis.*
Reporting on race, ethnicity, or other socially relevant groupings	*Please specify the socially constructed or socially relevant categorization variable(s) used in your manuscript and explain why they were used. Please note that such variables should not be used as proxies for other socially constructed/relevant variables (for example, race or ethnicity should not be used as a proxy for socioeconomic status). Provide clear definitions of the relevant terms used, how they were provided (by the participants/respondents, the researchers, or third parties), and the method(s) used to classify people into the different categories (e.g. self-report, census or administrative data, social media data, etc.) Please provide details about how you controlled for confounding variables in your analyses.*
Population characteristics	*Describe the covariate-relevant population characteristics of the human research participants (e.g. age, genotypic information, past and current diagnosis and treatment categories). If you filled out the behavioural & social sciences study design questions and have nothing to add here, write "See above."*
Recruitment	*Describe how participants were recruited. Outline any potential self-selection bias or other biases that may be present and how these are likely to impact results.*
Ethics oversight	*Identify the organization(s) that approved the study protocol.*

Note that full information on the approval of the study protocol must also be provided in the manuscript.

Field-specific reporting

Please select the one below that is the best fit for your research. If you are not sure, read the appropriate sections before making your selection.

☒ Life sciences ☐ Behavioural & social sciences ☐ Ecological, evolutionary & environmental sciences

For a reference copy of the document with all sections, see nature.com/documents/nr-reporting-summary-flat.pdf

Life sciences study design

All studies must disclose on these points even when the disclosure is negative.

Sample size	No formal statistical sample size calculation was performed for this study. Sample sizes were determined based on the number of domains from DUBs that could practically be produced and used as baits in phage display experiments, and further validated by biochemical assays.<br><br>For phage display selections and sequencing analyses, library complexity was set by the HD2 library design (see Benz et al., 2022). Sequencing depth were chosen to ensure sufficient coverage and reliable detection of enriched binders. For downstream validation experiments (e.g., affinity measurements, peptide spot arrays, and co-immunoprecipitation), sample sizes reflect standard experimental replication in the field and were sufficient to demonstrate consistent and reproducible interactions.<br><br>Key findings were confirmed across independent experiments and/or replicates, supporting the robustness of the results despite the absence of formal sample size estimation.
Data exclusions	Phage display: The analysis of the phage display data was filtered for medium/high confidence peptides, as defined using previously established quality metrics (see Benz et al., 2022). Low confidence peptides were excluded from the analysis to avoid corrupting the data due to deep sequencing.<br><br>For affinity measurements: outlier values were checked for potential pipetting errors and/or potential FITC contaminations in the unlabled peptide stocks. Experimental results were excluded if the quality controls failed, and experiments were repeated as needed.<br>No other predetermined exclusion criteria were established.
Replication	All experiments were performed in technical (phage display selections, affinity measurements) or biological (co-IPs) replicates
Randomization	No

Blinding	No

Reporting for specific materials, systems and methods

We require information from authors about some types of materials, experimental systems and methods used in many studies. Here, indicate whether each material, system or method listed is relevant to your study. If you are not sure if a list item applies to your research, read the appropriate section before selecting a response.

Materials & experimental systems

n/a	Involved in the study
☐	☒ Antibodies
☐	☒ Eukaryotic cell lines
☒	☐ Palaeontology and archaeology
☒	☐ Animals and other organisms
☒	☐ Clinical data
☒	☐ Dual use research of concern
☒	☐ Plants

Methods

n/a	Involved in the study
☒	☐ ChIP-seq
☒	☐ Flow cytometry
☒	☐ MRI-based neuroimaging

Antibodies

Antibodies used	Mouse Anti-Flag, Sigma F1804-50UG (Immunoprecipitation, co-IP, 1:2000; Rabbit Anti-HA, Cell Signalling Technology 3724S, 1:2000; Mouse Anti--tubulin (DM1A), Cell Signalling Technology 3873S, 1:2500; rabbit Anti-FLAG® M2 antibody (GST pulldown), Sigma Aldrich F3165-1MG, 1:5000; Mouse anti-M13 HRP-conjugated antibody, Nordic Biosite ARG66893-1, 1:5000; Anti-GST HRP Conjugate, Cytiva RPN1236, 1:3000 dilution; Rabbit Anti-GST Tag antibody (GST-pulldown, Sigma SAB4301139, 1:5000; Goat anti-mouse IRDye® 680RD, LI-COR 926-68070, 1:5000, Goat anti-rabbit IRDye® 800CW, LI-COR 926-32211, 1:5000.<br>Mouse HIF1A, R&D Systems Mab 1536, 1:2000; Rabbit CCP110, ProteinTech 12780-1-AP, 1:2500; Rabbit CEP192, ProteinTech 28700-1-AP, 1:2500; Rabbit ROBO1, ProteinTech 20219-1-AP, 1:2500; Rabbit GAPDH, Cell Signal Technology (CST) 2118S, 1:2500.
Validation	Antibodies were not validated as they are standard antibodies. TheM13 HRP conjugated antibody has been used in a large number of publications. The anti-GST antibody (Cytiva, RPN1236) is optimized for use in Western blotting with ECL Detection Reagents. The Monoclonal ANTI-FLAG® M2 is useful for identification and capture of FLAG® fusion proteins by common immunological procedures such as Western blots and<br>immuno-precipitation.

Eukaryotic cell lines

Policy information about cell lines and Sex and Gender in Research

Cell line source(s)	HEK293T cells, Sigma 85120602.<br>Human Embryonic Kidney 293 (HEK293) cells were obtained from MRC PPU cell culture facility.
Authentication	none
Mycoplasma contamination	Cell lines were not tested for mycoplasma
Commonly misidentified lines<br>(See ICLAC register)	No

Plants

Seed stocks	*Report on the source of all seed stocks or other plant material used. If applicable, state the seed stock centre and catalogue number. If plant specimens were collected from the field, describe the collection location, date and sampling procedures.*
Novel plant genotypes	*Describe the methods by which all novel plant genotypes were produced. This includes those generated by transgenic approaches, gene editing, chemical/radiation-based mutagenesis and hybridization. For transgenic lines, describe the transformation method, the number of independent lines analyzed and the generation upon which experiments were performed. For gene-edited lines, describe the editor used, the endogenous sequence targeted for editing, the targeting guide RNA sequence (if applicable) and how the editor was applied.*
Authentication	*Describe any authentication procedures for each seed stock used or novel genotype generated. Describe any experiments used to assess the effect of a mutation and, where applicable, how potential secondary effects (e.g. second site T-DNA insertions, mosiacism, off-target gene editing) were examined.*
