# Supplementary material for: Systematic discovery of motif-based interactions of the auxiliary domains of USP family deubiquitinases
Source: Nat Commun. 2026 May 18;17:6531. doi: 10.1038/s41467-026-73047-7 (PMC13376213; doi:10.1038/s41467-026-73047-7)
Supplement: Supplementary file 12 — Transparent Peer Review file [file 41467_2026_73047_MOESM12_ESM.pdf]

nature portfolio

Peer Review File

# Systematic Discovery of Motif-based Interactions of the Auxiliary Domains of USP Family Deubiquitinases

Corresponding Author: Professor Ylva Ivarsson

**This file contains all reviewer reports in order by version, followed by all author rebuttals in order by version.**

Version 0:

Reviewer comments:

Reviewer #1

(Remarks to the Author)
This work by Konstantinou et al. is a comprehensive and well-executed study that systematically examines the role of auxiliary domains (ADs) in the USP family of deubiquitinases (DUBs) in mediating protein–protein interactions through short linear motifs (SLiMs). The study provides a valuable resource for the ubiquitin research community and significantly advances our understanding of DUB substrate recognition. The manuscript presents high-quality data in identifying and validating AD–peptide interactions; however, it is less convincing in directly demonstrating how these interactions regulate enzymatic activity. Overall, the work reads more as a systematic survey—an extensive catalog of ADs within DUBs—than as a mechanistic study of their functional consequences.

## Noteworthy Results

The manuscript presents several noteworthy findings:
1. **Widespread SLiM-based Interactions:** The authors systematically screened 29 ADs from 19 USPs, revealing SLiM-based interactions for 14 of them. This demonstrates that motif-mediated targeting is a common, yet previously underappreciated, mechanism in this important enzyme family.
2. **Novel Motif Discovery:** The study successfully identifies and validates novel consensus binding motifs for several ADs. Notable examples include the `[KR]xxL[NED]` motif (UBIM) for the zf-UBP domains of USP20 and USP33, and the `[FW]x[IL]` motif for their DUSP2 domains.
3. **Multidomain Recognition:** A key finding is that DUBs such as CYLD, USP20, and USP33 possess multiple ADs that can bind distinct or shared peptide ligands. This supports a “multicomponent recognition” model in which combinatorial binding events contribute to substrate specificity and complex assembly.

## Significance and Originality
The work is significant and original. While previous studies have described individual examples of AD–SLiM interactions, this is the first large-scale, systematic effort to map them across the USP family. The findings shift the paradigm of DUB specificity from a sole focus on the catalytic domain and ubiquitin chain topology to a more integrated model that includes substantial regulation by auxiliary domains. The resulting dataset of interactions and motifs will be an invaluable resource, enabling the prediction of new DUB substrates and functions. The research is well-grounded in existing literature and uses the known USP7–p53 interaction as a robust validation of the approach.

## Support for Conclusions and Data Interpretation
The conclusions and claims are strongly supported by the data. The authors employ a combination of complementary techniques:
- **Discovery:** Proteomic peptide phage display (ProP-PD) for unbiased identification.
- **Validation:** Peptide SPOT arrays and fluorescence polarization (FP) for in vitro validation and affinity measurements.
- **Structural Insight:** State-of-the-art AlphaFold3 modeling to provide a structural basis for the observed interactions.
- **Cellular Context:** Co-immunoprecipitation (Co-IP) and IP–Mass Spectrometry (IP–MS) to confirm the interactions in a cellular setting.

The use of point mutations in both motifs and binding domains provides rigorous evidence for interaction specificity. The data are interpreted carefully. For example, the authors address the full-length HIF1A interaction with USP20, which appears

more complex than the identified SLiM alone, and propose plausible explanations such as multivalency—reflecting a nuanced understanding of the data.

## Methodological Soundness and Reproducibility
The methodology is sound and meets a very high standard in the field. The chosen techniques are appropriate for the research questions, and the Methods section provides sufficient detail to enable reproducibility. The data analysis pipelines, particularly for spectrometry data, are clearly described.

## Comments for Revision
The manuscript is of high quality and free of major flaws that would preclude publication. However, several points merit attention:

1. The authors rely heavily on AlphaFold for structural insights. Although these are high-confidence models based on current predictive standards, the reliability metrics used—ipTM and pLDDT—are less dependable for novel, low-homology protein–peptide interactions. The authors could strengthen their structural validation by including additional metrics such as Fnat (fraction of native contacts), ipSAE (interaction prediction score from aligned errors), and pDockQ (predicted DockQ).
2. The manuscript is dense with data, reflecting the impressive scope of the study, but a clearer visual summary (e.g., an overview schematic) might aid readability.
3. Inconsistency in Interaction Illustrations: The figures depicting AD–peptide interactions vary in style and detail. For example, Fig. 4E provides a detailed depiction with the peptide shown in green, the protein in orange, and key residue side chains shown as sticks, whereas Fig. 2A uses a gray protein and a green peptide without side-chain representation. A consistent visualization scheme would improve clarity.
4. The USP22 zf-UBP domain interaction with an ATXN7L1 peptide shows drastically different KD values for the three paralogs despite sequence conservation. This discrepancy is concerning because the authors emphasize the importance of conserved G..W..W residues, which do not explain the >160-fold difference in affinities. The authors should perform an orthogonal validation, such as isothermal titration calorimetry (ITC), to confirm the binding. Furthermore, this interaction should be contextualized with the known yeast Ubp8–Sgf73 interaction, homologous to USP22–ATXN7L1, within the yeast SAGA-DUB complex. The SAGA-DUB module requires four proteins to form a stable complex, and the zf-UBP domain interacts extensively with the other three components. The authors might consider modeling all four components rather than only the peptide and zf-UBP, as the current approach could produce misleading interpretations.

## Minor Typographical Errors

In several places, “UBP” is used as a shorthand for “zf-UBP domain.” The “zf” prefix should not be omitted, since “UBP” may refer to the catalytic ubiquitin protease domain (many yeast USPs are designated “UBPn”). Clarifying this usage will avoid confusion.

Reviewer #2

(Remarks to the Author)
Konstantinou et al. systematically probe the interaction of selected ubiquitin specific protease domains with linear peptide motifs by phage display using a library that samples intrinsically disordered regions from the human proteome. Out of 29 domains and two full-length proteins 14 domains from nine different proteases were identified to bind such motifs. Selected peptide-protein interactions are further investigated by FP assays with synthetic peptides, structure prediction and in some cases mutational analysis, alanine scanning and immunoprecipitation with the cellular binding partner. Key novel findings include the identification of common motifs for binding to the USP20 and USP33 zinc finger domains with similar affinities, while upon further investigation the DUSP domains share a common motif but displayed somewhat greater selectivity, notably for transcription factor HIF1A. Furthermore, novel insights into the interactions of the SAGA complex associated USP22 zinc finger domain were gained and all three of the CAP-Gly domains of CYLD were found to interact with linear peptide motifs. Several other interactions were mapped for the first time.
While linear binding motif interactions with a USP auxiliary domain identified by phage display have been reported before (doi: 10.1007/978-1-0716-2803-4_12; doi: 10.1074/jbc.H118.006803), this work’s scope is impressive and systematically probes a large proportion of USP auxiliary domains, many of which have not previously been known to interact with the identified binding partners. The large-scale identification of these USP binding motifs is a valuable contribution to the field, provides new insights into the interactome and function of several USPs by association, and will provide a rich resource for further study. The study is technically sound and the data interpretation valid.
I only have minor comments:
Description of AlphaFold structural models: Descriptions of these models should clearly indicate that these are predictions: please replace terms such as AF3 model ” illustrates/reveals” with “predicts/suggests” as appropriate throughout. High confidence predictions are still not proof that the binding modes are correct (especially the detail).
Line 81: please include information on adjacent residues from phage protein
Line 183: Statement that UBL domains are known to bind UIMs is only true for a subset, please amend.
Line 212: this should be USP11 not USP19?
Discussion: please include sentence that interactions could be modulated in full-length proteins where this has not been investigated.
Line 478: comparison with degrons: this is quite speculative, so should be labelled as such
Line 602: please provide information on whether peptides for FP assays were N or C-terminally labelled and whether any spacers were used.

Reviewer #3

(Remarks to the Author)
The work entitled "Systematic Discovery of Motif-based Interactions of the Auxiliary Domains of USP Family Deubiquitinases" presents an impressive and comprehensive study on the recognition mechanisms that underlie substrate specificity within the USP family of deubiquitinases (DUBs).
Through a systematic investigation of 29 auxiliary domains (ADs) and two full-length USPs, the authors identify short linear motif (SLiM)-based interactions that contribute to substrate targeting and complex assembly. The study stands out for the extensive and well-designed experimental work, combining peptide-phage display, peptide arrays, and affinity assays to validate multiple novel AD–SLiM interactions. The figures are exceptionally clear and well-structured, effectively guiding the reader through complex datasets and models. The proteins investigated—such as CYLD, USP11, USP19, USP20, USP22, and USP33—are highly relevant due to their central roles in protein degradation, signaling, and complex assembly.
Importantly, the findings reveal that SLiM-binding is a widespread feature among USP auxiliary domains, offering new insights into how these enzymes achieve specificity beyond ubiquitin-chain recognition. The discovery of redundant and cooperative binding modes, particularly within USP20 and USP33, highlights sophisticated regulatory strategies and opens new avenues for exploring DUB-mediated control of cellular pathways.
Overall, this is a rigorous and conceptually significant contribution that not only broadens our understanding of DUB function but also provides valuable tools for future applications such as targeted protein stabilization via DUBTAC strategies.
Therfore I would like to recommend the work for publication in Nature Communcation since I believe it is a great work which makes a significant contribution to the protein-SliM Interaction fiedl. Nonetheless I have some moderate suggestions related to how the authors contextualize and reach out to the broader field of SliMs with their results. Specific comments follow:

1) The work is organized in six sections wch with one figure focusing on a specific set of proteins and their SLiM-based interactions. After all sections the work goes directly o the Concluding remarks. I think the work would benefit a lot, if a new section, perhaps a with a Table, is added where the findings are summarized. Specifcically, which protein / domain has been found to bind which new SliM and what are the identified target proteins.

2) I think work needs to be contextualized in the wealth of knowledge on SliMs. In particular authors correctly cite the ELM database (Kumar, M. et al. ELM-the Eukaryotic Linear Motif resource-2024 update. Nucleic Acids Res 52, D442-D455 (2024). https://doi.org:10.1093/nar/gkad1058 ) , but in the introduction. I think it would be good to mention whether or not the new found SliMs are new or were already repoted in ELM.

3) Another missed point is the relation of SliMs and the proteins that display them and their relation to disease, as for example can be found in OMIM and Uniprot. It has been recently shown (https://doi.org/10.1371/journal.pcbi.1012829) that pathogenic variants found in the SliMs can help to characterize SliMs and predict new proteins harboring the,. Perhaps the authors could comment on whether the new found SlIms and their host proteins are (or not) related to disease and display what type of variants.

4) Finally, it would be nice if once the new SliMs are defined by their regular expression, to conduct a fast search in Uniprot and provide the potential number of potential proteins harboring these SliMs are present in the human proteome.

Version 1:

Reviewer comments:

Reviewer #1

(Remarks to the Author)
The revised manuscript has adequately addressed all the comments I previously made. Although the manuscript is lengthy, it represents a rich and valuable resource for the ubiquitin research community. I recommend that it be accepted for publication.

There are only two minor typographical issues that should be corrected:
1. Inconsistent spelling of “ubiquitylation” vs. “ubiquitination” and “deubiquitylation” vs. “deubiquitination.” For example: “ubiquitylated” on line 39 and “ubiquitinated” on line 553.
2. An extra “domains” after “ADs” on line 79.

These minor corrections can be addressed during the typesetting stage and do not require the manuscript to be sent back to the reviewers.

Reviewer #3

(Remarks to the Author)
he revised version of the ms entitled "Systematic Discovery of Motif-based Interactions of the Auxiliary Domains of USP Family Deubiquitinases" has been carefully reviewed. I am pleased to inform you that the authors have successfully addressed all my concerns, and the manuscript is suitable in my opinion to be accepted for publication in Nature

Communications.

The updates provided in this revision significantly enhance the clarity and impact of the study.
Specifically, the following changes have strengthened the work:

Consolidated Data Synthesis: The addition of Table 1 effectively summarizes the newly defined SLiMs, motifs, and target proteins, providing a high-utility reference for the field.

Disease Contextualization: By exploring the overlap between the identified SLiMs and pathogenic mutations (e.g., in ELOVL4 and MYH11), authors have successfully bridged the gap between biochemical discovery and clinical relevance. As a final minor suggestion to further enrich the discussion on pathogenic variants, I recommend adding the following reference: doi: 10.1371/journal.pcbi.1012829 , where it is shown that pathogenic variants found in the SLiMs can help to characterize SLiMs and predict new proteins harboring them.

Proteome-Wide Perspective: The inclusion of comparisons to the ELM database and the discussion on SLiMSearch provides necessary context for the broader protein-SLiM interaction community.

As seen in the revised version and response letter, the auhtors also performed most of the requests by the other 2 reviewers.

**Open Access** This Peer Review File is licensed under a Creative Commons Attribution 4.0 International License, which permits use, sharing, adaptation, distribution and reproduction in any medium or format, as long as you give appropriate credit to the original author(s) and the source, provide a link to the Creative Commons license, and indicate if changes were made.
In cases where reviewers are anonymous, credit should be given to 'Anonymous Referee' and the source.
The images or other third party material in this Peer Review File are included in the article’s Creative Commons license, unless indicated otherwise in a credit line to the material. If material is not included in the article’s Creative Commons license and your intended use is not permitted by statutory regulation or exceeds the permitted use, you will need to obtain permission directly from the copyright holder.
To view a copy of this license, visit https://creativecommons.org/licenses/by/4.0/

**REPLY to REVIEWER COMMENTS**

**Reviewer #1 (Remarks to the Author):**

This work by Konstantinou et al. is a comprehensive and well-executed study that systematically examines the role of auxiliary domains (ADs) in the USP family of deubiquitinases (DUBs) in mediating protein–protein interactions through short linear motifs (SLiMs). The study provides a valuable resource for the ubiquitin research community and significantly advances our understanding of DUB substrate recognition. The manuscript presents high-quality data in identifying and validating AD–peptide interactions; however, it is less convincing in directly demonstrating how these interactions regulate enzymatic activity. Overall, the work reads more as a systematic survey—an extensive catalog of ADs within DUBs—than as a mechanistic study of their functional consequences.

## Noteworthy Results

The manuscript presents several noteworthy findings:
1. **Widespread SLiM-based Interactions:** The authors systematically screened 29 ADs from 19 USPs, revealing SLiM-based interactions for 14 of them. This demonstrates that motif-mediated targeting is a common, yet previously underappreciated, mechanism in this important enzyme family.
2. **Novel Motif Discovery:** The study successfully identifies and validates novel consensus binding motifs for several ADs. Notable examples include the `[KR]xxL[NED]` motif (UBIM) for the zf-UBP domains of USP20 and USP33, and the `[FW]x[IL]` motif for their DUSP2 domains.
3. **Multidomain Recognition:** A key finding is that DUBs such as CYLD, USP20, and USP33 possess multiple ADs that can bind distinct or shared peptide ligands. This supports a “multicomponent recognition” model in which combinatorial binding events contribute to substrate specificity and complex assembly.

## Significance and Originality
The work is significant and original. While previous studies have described individual examples of AD–SLiM interactions, this is the first large-scale, systematic effort to map them across the USP family. The findings shift the paradigm of DUB specificity from a sole focus on the catalytic domain and ubiquitin chain topology to a more integrated model that includes substantial regulation by auxiliary domains. The resulting dataset of interactions and motifs will be an invaluable resource, enabling the prediction of new DUB substrates and functions. The research is well-grounded in existing literature and uses the known USP7–p53 interaction as a robust validation of the approach.

## Support for Conclusions and Data Interpretation
The conclusions and claims are strongly supported by the data. The authors employ a combination of complementary techniques:
- **Discovery:** Proteomic peptide phage display (ProP-PD) for unbiased identification.
- **Validation:** Peptide SPOT arrays and fluorescence polarization (FP) for in vitro validation and affinity measurements.
- **Structural Insight:** State-of-the-art AlphaFold3 modeling to provide a structural basis for the observed interactions.
- **Cellular Context:** Co-immunoprecipitation (Co-IP) and IP–Mass Spectrometry (IP–MS) to confirm the interactions in a cellular setting.

The use of point mutations in both motifs and binding domains provides rigorous evidence for interaction specificity. The data are interpreted carefully. For example, the authors address the full-length HIF1A interaction with USP20, which appears more complex than the identified SLiM alone, and propose plausible explanations such as multivalency—reflecting a nuanced understanding of the data.

## Methodological Soundness and Reproducibility
The methodology is sound and meets a very high standard in the field. The chosen techniques are appropriate for the research questions, and the Methods section provides sufficient detail to enable reproducibility. The data analysis pipelines, particularly for spectrometry data, are clearly described.

## Comments for Revision
The manuscript is of high quality and free of major flaws that would preclude publication.

**Reply:** We thank the reviewer for appreciating the quality of our study.

However, several points merit attention:

**Comment 1**. The authors rely heavily on AlphaFold for structural insights. Although these are high-confidence models based on current predictive standards, the reliability metrics used—ipTM and pLDDT—are less dependable for novel, low-homology protein–peptide interactions. The authors could strengthen their structural validation by including additional metrics such as Fnat (fraction of native contacts), ipSAE (interaction prediction score from aligned errors), and pDockQ (predicted DockQ).

**Reply:** We appreciate the reviewer's concern. However, multiple benchmarking studies, including our own previous work (Lee, Hubrich and Varga et al.), have demonstrated that ipTM and the derived confidence score (0.8×ipTM + 0.2×pTM) already indicate model quality well for peptide-protein interactions regardless of homology level, as peptides are rarely covered by the input MSA. Indeed, a recent systematic study specifically investigated the role of MSAs and training set homology in peptide-protein docking (Guan & Keating, 2025) and found that the peptide MSA has minimal impact on prediction accuracy for most complexes, while also confirming that ipTM+pTM correlates well with prediction quality (Pearson >0.7), outperforming pDockQ.

The alternative metrics suggested - ipSAE and pDockQ - are derived from the same underlying AlphaFold confidence data (predicted aligned error and pLDDT) as ipTM. They would therefore not provide independent validation nor address homology-related concerns if such concerns were valid. Fnat (fraction of native contacts) requires an experimentally determined reference structure for comparison and is thus not applicable to predictions of novel interactions.

That being said, flexible regions flanking binding motifs can lower ipTM scores even when the core interaction is well-predicted. To address this, we developed actifpTM (see Varga et al., 2025 for background), to focus scoring on the structured interface while discounting disordered flanking regions. Consistent with this notion, we found that the actifpTM scores were systematically higher than the ipTM scores for the modelled complexes (see below). Importantly, we validate predictions experimentally through alanine scanning, providing orthogonal confirmation of the modeled interactions regardless of predicted confidence.

| AF3 model | ipTM score | actifpTM |
|---|---|---|
| cyld_cap-gly_1_8-210_rbm25_558-573 | 0.93 | 0.96 |
| cyld_cap-gly_2_235-307_fbxo46_168-183 | 0.8 | 0.87 |
| cyld_cap-gly_3_460-566_hectd4_3003-3018 | 0.78 | 0.92 |
| usp11_ubl_207-284_znf518a_679-694 | 0.7 | 0.94 |

| | | |
|---|---|---|
| usp11_ubl_207-284_zwint_202-219 | 0.71 | 0.79 |
| usp19_ubl_679-766_hook2_590-605 | 0.78 | 0.84 |
| usp19_ubl_679-766_wwc1_1029-1044 | 0.8 | 0.84 |
| usp20_ubp_1-99_apba2_327-338 | 0.87 | 0.94 |
| usp20_ubp_1-99_robo1_1494-1506 | 0.7 | 0.88 |
| usp22_zf-ubp_37-130_atxn7l1_36-51 | 0.73 | 0.82 |
| usp22_zf-ubp_37-130_atxn7l1_36-57 | 0.83 | 0.92 |
| usp25_nn_745-1055_hbs1l_309-324 | 0.6 | 0.92 |
| usp33_dusp2_806-921_hif1a_564-579 | 0.74 | 0.89 |
| usp33_ubp_36-130_apba2_327-338 | 0.83 | 0.93 |
| usp33_ubp_36-130_robo1_1494-1506 | 0.66 | 0.85 |
| usp7_math_53-205_mdc1_1202-1217 | 0.77 | 0.96 |
| usp7_math_63-202_map1b_1726-1741 | 0.82 | 0.96 |
| usp7_math_63-202_peg3_1542-1557 | 0.83 | 0.96 |
| usp20_dusp2_gpr137 | 0.77 | 0.94 |
| usp20_dusp2_hif1a | 0.7 | 0.9 |
| usp33_dusp2_gpr137b | 0.72 | 0.9 |
| usp33_dusp2_hif1a | 0.7 | 0.86 |

The information has now been added to the supplemental information (Supp. Table 7). We have also added the following section to the discussion:

*"We explored the found interactions using AF3 and validated the models by mutagenic analysis. Modelling domains and peptide fragments rather than full-length proteins have been shown to improve prediction accuracy in AlphaFold-based approaches[58,59]. Several of the domain–peptide interactions found here were modelled with high confidence using AF3, based on ipTM scores. Using the actifpTM score[68] which focus scoring on the structured interface while discounting disordered flanking region, the confidences of the models were even higher (Supp. Table 7). However, the precise structural details of these interactions within the context of the full-length protein complexes remain to be established experimentally. It remains possible that additional contacts contribute to complex formation in the full-length proteins, or that the structural context of the identified motifs within the intact proteins may impede binding."*

2. The manuscript is dense with data, reflecting the impressive scope of the study, but a clearer visual summary (e.g., an overview schematic) might aid readability.

**Reply:** Thank you for the suggestion. We have updated the schematic in Fig 1A and Fig 1C, which we hope improve the overview of the study. Following the suggestion by reviewer 3 we have also added a final table that summarizes the motifs discovered.

3. Inconsistency in Interaction Illustrations: The figures depicting AD–peptide interactions vary in style and detail. For example, Fig. 4E provides a detailed depiction with the peptide shown in green, the protein in orange, and key residue side chains shown as sticks, whereas Fig. 2A uses a gray protein and a green peptide without side-chain representation. A consistent visualization scheme would improve clarity.

**Reply:** We agree. We have now polished the figures and ensured that the representations of the modelled complexes more consistent.

4. The USP22 zf-UBP domain interaction with an ATXN7L1 peptide shows drastically different KD values for the three paralogs despite sequence conservation. This discrepancy is concerning because the authors emphasize the importance of conserved G..W..W residues, which do not explain the >160-fold difference in affinities. The authors should perform an orthogonal validation, such as isothermal titration calorimetry (ITC), to confirm the binding.

**Reply:** This is a fair point.

However, the reason for the affinity differences are not to be found in the method used to determine affinities, but in the actual peptides used for the experiments. The main purpose of the affinity measurement with the AXTN7 and ATXN7L2 peptides were to validate that the binding region is conserved. When designing the peptides, we chose to use 16 amino acid peptides, which cover the main motif. However, these peptides are five amino acids shorter than the ATXN7L1 peptide used for the measurement, and although the results confirm that both the AXTN7 and ATXN7L2 peptides do bind USP22 zf-UBP domain, the affinity data are not directly comparable . The differences in peptide lengths used were highlighted in the previous Fig. 6F, where the sequences of the peptides used are underlined.

$ATXN7_{84\text{-}105}$ LPSPEVMLGQSWNLWVEASKLP
$ATXN7L1_{36\text{-}57}$ VPSPEAFLGKPWSSWIDAAKLH
$ATXN7L2_{18\text{-}39}$ VPSLDDFAGQSWSSWVERADLP
* * *

To support this notion, we obtained ATXN1L1 and ATX7NL2 peptides of two different lengths and determined the affinities using the FP based approach. Consistent with the hypothesis, we found the extensions of the peptides to increase the affinities considerably (between 6-100 times). See panel C and F in Figure 6.

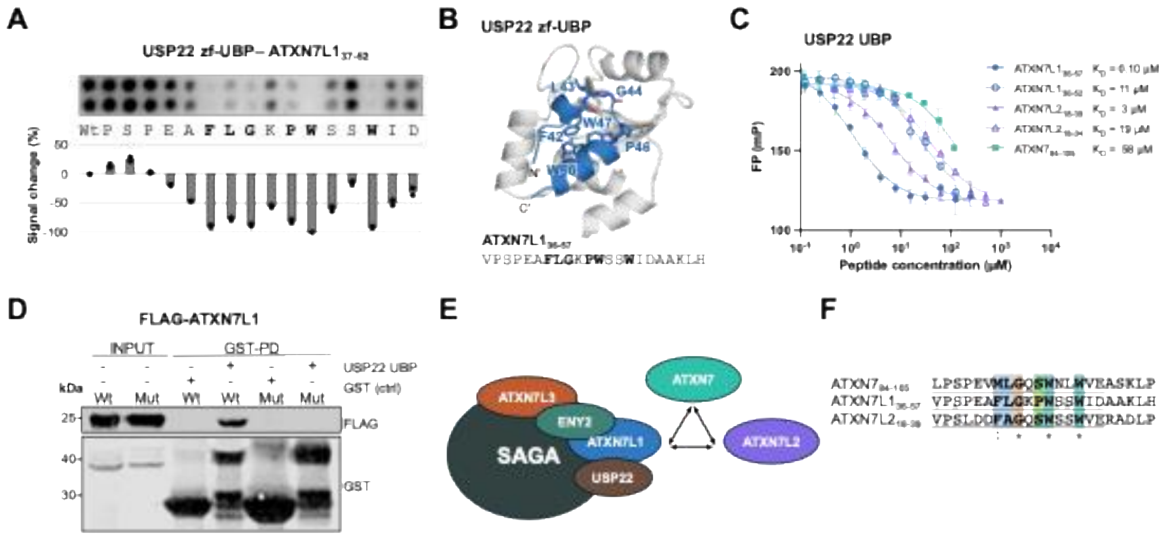

Thus, while the motif defined by the spot array is necessary for binding, the C-terminal flanking region is contributing to the affinity.

Regarding the use of an alternative method (e.g. ITC) for affinity determination, we have already in previous studies shown the Kd values obtained from the competitive FP assay compare very well to Kd values determined by ITC, see for example Sundell et al., 2018 (PMID: 30126976) and Kliche et al, 2024 (PMID: **39009827**).

5. Furthermore, this interaction should be contextualized with the known yeast Ubp8–Sgf73 interaction, homologous to USP22–ATXN7L1, within the yeast SAGA-DUB complex. The SAGA-DUB module requires four proteins to form a stable complex, and the zf-UBP domain interacts extensively with the other three components. The authors might consider modeling all four components rather than only the peptide and zf-UBP, as the current approach could produce misleading interpretations.

**Reply.** This is correct. Following the suggestion by the reviewer we modelled the complex consisting of full length ATXN7L1, USP22, ENY2 and ATXN7L3. Reassuringly, the predicted interaction site between the ATXN7L1 motif-USP22 UBL domain were found to be virtually identical in the two models, see below. We have added the information to the Supplemental Figure 6.

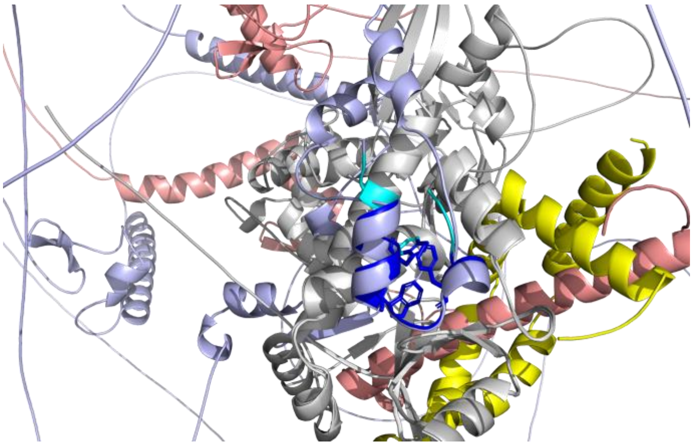

**Supplemental Fig. 6.** AF3 model of the SAGA complex (full-length ATXN7L1 (light blue), USP22 (grey), ENY2 (yellow)and ATXN7L3 (light red)), overlayed with complex of USP22 zf-UBL-ATXN7L1 (colored by the confidence of the AF3 model). Reassuringly, the predicted interaction site between the ATXN7L1 motif-USP22 UBL domain were found to be virtually identical in the two models.

## Minor Typographical Errors

In several places, “UBP” is used as a shorthand for “zf-UBP domain.” The “zf” prefix should not be omitted, since “UBP” may refer to the catalytic ubiquitin protease domain (many yeast USPs are designated “UBPn”). Clarifying this usage will avoid confusion.

**Reply:** Thanks for pointing this out. We have replaced “UBP” with “zf-UBP” throughout the text and figures.

**Reviewer #2 (Remarks to the Author):**

Konstantinou et al. systematically probe the interaction of selected ubiquitin specific protease domains with linear peptide motifs by phage display using a library that samples intrinsically disordered regions from the human proteome. Out of 29 domains and two full-length proteins 14 domains from nine different proteases were identified to bind such motifs. Selected peptide-protein interactions are further investigated by FP assays with synthetic peptides, structure prediction and in some cases mutational analysis, alanine scanning and immunoprecipitation with the cellular binding partner. Key novel findings include the identification of common motifs for binding to the USP20 and USP33 zinc finger domains with similar affinities, while upon further investigation the DUSP domains share a common motif but displayed somewhat greater selectivity, notably for transcription factor HIF1A. Furthermore, novel insights into the interactions of the SAGA complex associated USP22 zinc finger domain were gained and all three of the CAP-Gly domains of CYLD were found to interact with linear peptide motifs. Several other interactions were mapped for the first time.
While linear binding motif interactions with a USP auxiliary domain identified by phage display have been reported before (doi: 10.1007/978-1-0716-2803-4_12; doi: 10.1074/jbc.H118.006803), this work's scope is impressive and systematically probes a large proportion of USP auxiliary domains, many of which have not previously been known to interact with the identified binding partners. The large-scale identification of these USP binding motifs is a valuable contribution to the field, provides new insights into the interactome and function of several USPs by association, and will provide a rich resource for further study. The study is technically sound and the data interpretation valid.

**Reply:** We thank the reviewer for appreciating our study.

I only have minor comments:

Description of AlphaFold structural models: Descriptions of these models should clearly indicate that these are predictions: please replace terms such as AF3 model " illustrates/reveals" with "predicts/suggests" as appropriate throughout. High confidence predictions are still not proof that the binding modes are correct (especially the detail).

**Reply:** We agree and have made the requested replacements throughout the manuscript.

Line 81: please include information on adjacent residues from phage protein

**Reply:** We have added the following information "flanked by glycine-serine linkers (SSSG- 16 aa peptide-GGGSGG)".

Line 183: Statement that UBL domains are known to bind UIMs is only true for a subset, please amend.

**Reply:** We agree and have made this clear. The text now reads ".Ubiquitin and some UBL domains are known to bind the ubiquitin interacting motif (UIM; [DE]x{2,4}[AV]xx[LMIV]xx) that forms an α-helix upon binding[27,28] and variants thereof [29,30].

Line 212: this should be USP11 not USP19?

**Reply:** This is correct. Thank you for noticing the mistake.

Discussion: please include sentence that interactions could be modulated in full-length proteins where this has not been investigated.

**Reply:** We appreciate the suggestion, and have the following to the discussion

*"Modelling domains and peptide fragments rather than full-length proteins have been shown to improve prediction accuracy in AlphaFold-based approaches[58,59]. Several of the domain–peptide interactions found here were modelled with high confidence using AF3, based on ipTM scores. Using the actifpTM score[68] which focus scoring on the structured interface while discounting disordered flanking region, the confidences of the models were even higher (Supp. Table 7). However, the precise structural details of these interactions within the context of the full-length protein complexes remain to be established experimentally. It remains possible that additional contacts contribute to complex formation in the full-length proteins, or that the structural context of the identified motifs within the intact proteins may impede binding."*

Line 478: comparison with degrons: this is quite speculative, so should be labelled as such

**Reply:** We agree, this is a speculation. We have now made this clear. The text now reads "*These interactions may contribute to complex and potentially substrate recognition analogous to the recognition of degrons by E3 ligases."*

Line 602: please provide information on whether peptides for FP assays were N or C-terminally labelled and whether any spacers were used.

**Reply:** We have clarified the labeling in the method section.

"*FITC-labelled peptides (N-terminally FITC labelled without spacer and C-terminally amidated) were dissolved in dimethyl sulfoxide (DMSO) and unlabelled peptides (N-terminally acetylated and C-terminally amidated) were dissolved in 50 mM phosphate buffer pH 7.4*."

Reviewer #3 (Remarks to the Author):

The work entitled "Systematic Discovery of Motif-based Interactions of the Auxiliary Domains of USP Family Deubiquitinases" presents an impressive and comprehensive study on the recognition mechanisms that underlie substrate specificity within the USP family of deubiquitinases (DUBs).
Through a systematic investigation of 29 auxiliary domains (ADs) and two full-length USPs, the authors identify short linear motif (SLiM)-based interactions that contribute to substrate targeting and complex assembly. The study stands out for the extensive and well-designed experimental work, combining peptide-phage display, peptide arrays, and affinity assays to validate multiple novel AD–SLiM interactions. The figures are exceptionally clear and well-structured, effectively guiding the reader through complex datasets and models. The proteins investigated—such as CYLD, USP11, USP19, USP20, USP22, and USP33—are highly relevant due to their central roles in protein degradation, signaling, and complex assembly. Importantly, the findings reveal that SLiM-binding is a widespread feature among USP auxiliary domains, offering new insights into how these enzymes achieve specificity beyond ubiquitin-chain recognition. The discovery of redundant and cooperative binding modes, particularly within USP20 and USP33, highlights sophisticated regulatory strategies and opens new avenues for exploring DUB-mediated control of cellular pathways.
Overall, this is a rigorous and conceptually significant contribution that not only broadens our understanding of DUB function but also provides valuable tools for future applications such as targeted protein stabilization via DUBTAC strategies.
Therefore I would like to recommend the work for publication in Nature Communcation since I believe it is a great work which makes a significant contribution to the protein-SliM Interaction field.

**Reply:** We thank the reviewer for appreciating our study.

Nonetheless I have some moderate suggestions related to how the authors contextualize and reach out to the broader field of SliMs with their results. Specific comments follow:

**Comment**
1) The work is organized in six sections each with one figure focusing on a specific set of proteins and their SLiM-based interactions. After all sections the work goes directly to the Concluding remarks. I think the work would benefit a lot, if a new section, perhaps a with a Table, is added where the findings are summarized. Specifically, which protein / domain has been found to bind which new SliM and what are the identified target proteins.

**Reply**

Thank you for the suggestion. While there was a summary in Fig. 1 we realize from the reviewers' comments that the placement of the summary is better towards the end of the manuscript. We have therefore added a table to the Discussion:

Almost all of the found motifs were defined for the first time in the current study (Table 1), and thus not previously reported in for example the ELM database[5].

**Table 1. Overview of SLiMs (re)defined in the current study**

| Gene | Domain | SLiM | Motif name | Previously described |
|---|---|---|---|---|
| CYLD | CAP-Gly_1 | LxRMxxxAxxRR | CYLD CG1IM | |

| | | | | |
|---|---|---|---|---|
| USP7 | MATH | [AP][GS]TS | Variant of DOC_USP7_MATH_1 | 16, 5 |
| USP11 | UBL | Fxx[LF][LVF] | USP11 UIM_1 | 13 |
| USP11 | UBL | [LFY]PxW[IW] | USP11 UIM_2 | |
| USP19 | UBL | LRx{3,4}R[FY] | USP19 UIM | |
| USP20/USP33 | zf-UBP | [KR]xxL[NED] | USP20/33 UBPIM | |
| USP20/USP33 | DUSP2 | [FW]x[IL] | USP20/33 DUSPIM | |
| USP22 | UBP | [FM][AL]Gx[PS]WxxWx | USP22 UBPIM | |
| USP25 | CTD | WxL | USP25 CTDIM | |

2) I think work needs to be contextualized in the wealth of knowledge on SliMs. In particular authors correctly cite the ELM database (Kumar, M. et al. ELM-the Eukaryotic Linear Motif resource-2024 update. Nucleic Acids Res 52, D442-D455 (2024). https://doi.org:10.1093/nar/gkad1058 ) , but in the introduction. I think it would be good to mention whether or not the new found SliMs are new or were already repoted in ELM.

**Reply**

Thank you for the suggestion. Most of the found motifs are defined for the first time in the current study. We have added this to the Discussion. We have also added the following to the introduction:

"*We identified potential peptide ligands for 15 of the protein baits. For 9 of the domains, we defined consensus binding motifs through an integrated approach combining ProP-PD data, structural modelling, and peptide SPOT array alanine scanning. Most (7 out 9) of these consensus motifs are newly defined in this study*."

3) Another missed point is the relation of SliMs and the proteins that display them and their relation to disease, as for example can be found in OMIM and Uniprot. It has been recently shown (https://doi.org/10.1371/journal.pcbi.1012829) that pathogenic variants found in the SliMs can help to characterize SliMs and predict new proteins harboring them. Perhaps the authors could comment on whether the new found SlIms and their host proteins are (or not) related to disease and display what type of variants.

**Reply**

Thank you for the suggestion. To contextualize the results, we

1. Added information on DUBs and diseases to the Discussion:

*"Finally, several SLiM-binding DUBs are associated with human disease, reflecting their central roles in regulating protein stability via deubiquitination across multiple signalling pathways. For example, mutations in CYLD and USP7 are linked to familial cylindromatosis[61] and Hao–Fountain syndrome[62], respectively. In addition, other DUBs, including USP11 and USP20, have been implicated in tumorigenesis and are being explored as potential therapeutic targets[34,63]. The novel peptide ligands could thus potentially be explored for specifically targeting the interactions of the binding DUBs."*

2. Explored the overlap between reported disease mutations and found SLiMs, which revealed that there are only three pathogenic mutations that hit directly on the found consensus motifs.
    a. A pathogenic W246G mutation in a loop region between two transmembrane domains of ELOVL4 hits directly on the found binding motif of USP11 DUSP-UBL domain. The pathogenic mutation is associated with Spinocerebellar ataxia type 34, and seemed interesting at first. However, this region is predicted

to be in the ER lumen, and thus less likely to be binding available (ref PMID: 31616255).

b. Pathogenic mutations of Myosin-11 I (R1275L and R1275Q), which are linked to aortic aneurysm, familial thoracic 5, affects a key residue of the USP20 zf-UBP binding motif. The mutations are in a coiled-coil region of Myosin-11 and are known to affect the filament assembly (PMID: 16444274**).**

We have added a section on this to the discussion.

*"Related to disease associations, we note that only a small number of the identified SLiMs that bind auxiliary domains of USPs are directly affected by pathogenic mutations targeting key motif residues (Supp. Table 2). Specifically, we identified only three such mutations: a pathogenic ELOVL4 W246G variant affecting the putative USP11-binding site, and two pathogenic MYH11 variants (R1275L and R1275Q) that disrupt the putative USP20 UBIM. However, in the case of ELOVL4 W246G, the affected region is located within an ER luminal domain, rendering a direct interaction with cytosolic USPs unlikely. Moreover, the MYH11 R1275L/Q variants are reported to exert their pathogenic effects primarily by disrupting myosin filament assembly, rather than by altering protein stability or regulatory interactions[64]. Taken together, these observations suggest that SLiMs engaging auxiliary domains of DUBs are rarely targeted directly by disease-associated mutations, in comparison to degrons, which appear to be more frequently perturbed in human disease[65,66]."*

4) Finally, it would be nice if once the new SliMs are defined by their regular expression, to conduct a fast search in Uniprot and provide the potential number of potential proteins harboring these SliMs are present in the human proteome.

Reply: In theory this would make a nice addition, but in practice it may be misleading. There is a large overprediction of binding sites based on simple motif scanning of protein sequences, especially if run on Uniprot sequences without taking into account if regions are intrinsically disordered or not. Thus, without the pairing with experimental validations we refrain from adding this analysis. However, we added a comment on this to the discussion, and suggest a suitable approach to follow for the interested reader.

'

"The study thus opens doors for follow up studies on here identified interactions, as well as the use of the defined motifs for scanning for binding sites within the intrinsically disordered regions of interaction partners and putative substrates using for example SLiMSearch[39]. Given the high false positive rates of such motif-based predictions, the analysis should be paired with experimental validations (for example of computational and experimental pipeline see[57]). Here, we used such approach for uncovering additional binding sites for the zf-UBP and DUSP2 domains of USP20 and USP33 (Fig. 4G and 5L). "

We thank the reviewers for insightful and helpful comments and suggestions.

**Reply to reviewers:**

**Reviewer #1 (Remarks to the Author):**

The revised manuscript has adequately addressed all the comments I previously made. Although the manuscript is lengthy, it represents a rich and valuable resource for the ubiquitin research community. I recommend that it be accepted for publication.

There are only two minor typographical issues that should be corrected:
1. Inconsistent spelling of “ubiquitylation” vs. “ubiquitination” and “deubiquitylation” vs. “deubiquitination.” For example: “ubiquitylated” on line 39 and “ubiquitinated” on line 553.
2. An extra “domains” after “ADs” on line 79.

These minor corrections can be addressed during the typesetting stage and do not require the manuscript to be sent back to the reviewers.

**Reply:**

Thank you for the feedback. We have replaced ”ubiquitylation” with “ubiquitination” throughout the manuscript and removed the extra ”domain” on line 79.

**Reviewer #3 (Remarks to the Author):**
The revised version of the ms entitled "Systematic Discovery of Motif-based Interactions of the Auxiliary Domains of USP Family Deubiquitinases" has been carefully reviewed. I am pleased to inform you that the authors have successfully addressed all my concerns, and the manuscript is suitable in my opinion to be accepted for publication in Nature Communications.

The updates provided in this revision significantly enhance the clarity and impact of the study.Specifically, the following changes have strengthened the work:

Consolidated Data Synthesis: The addition of Table 1 effectively summarizes the newly defined SLiMs, motifs, and target proteins, providing a high-utility reference for the field.

Disease Contextualization: By exploring the overlap between the identified SLiMs and pathogenic mutations (e.g., in ELOVL4 and MYH11), authors have successfully bridged the gap between biochemical discovery and clinical relevance. As a final minor suggestion to further enrich the discussion on pathogenic variants, I recommend adding the following reference: doi: 10.1371/journal.pcbi.1012829 , where it is shown that pathogenic variants found in the SLiMs can help to characterize SLiMs and predict new proteins harboring them.

Proteome-Wide Perspective: The inclusion of comparisons to the ELM database and the discussion on SLiMSearch provides necessary context for the broader protein-SLiM interaction community.

As seen in the revised version and response letter, the authors also performed most of the requests by the other 2 reviewers.

**Reply:**

Thank you for the feedback and the appreciation of the revised manuscript. While we appreciate the suggested reference, we have already reached the maximum number of references allowed. As the paper is not critical for the discussion, we thus respectfully sustain from adding more references to avoid removing other references.

--

We thank the reviewers for their valuable comments during the revision process which has contributed to the quality of the study.
